# Advocating for Precision Neoadjuvant and Subsequent Adjuvant Therapies in Esophageal Squamous Cell Carcinoma: Evidence From a Real‐World Multicenter Study

**DOI:** 10.1002/mco2.70909

**Published:** 2026-08-14

**Authors:** Peiyu Wang, Xiufeng Wei, Yongkui Yu, Zhiqian Zhao, Qiuchen Ge, Jianhong Lian, Wenqun Xing, Xiangnan Li, Yin Li

**Affiliations:** ^1^ Department of Thoracic Surgery The First Affiliated Hospital of Zhengzhou University Zhengzhou Henan China; ^2^ Department of Thoracic Surgical Oncology National Cancer Center/Cancer Hospital, Chinese Academy of Medical Sciences and Peking Union Medical College Beijing China; ^3^ Department of Thoracic Surgery The Affiliated Cancer Hospital of Zhengzhou University, Henan Cancer Hospital Zhengzhou Henan China; ^4^ Department of Thoracic Surgery Shanxi Province Cancer Hospital/Shanxi Hospital Affiliated to Cancer Hospital, Chinese Academy of Medical Sciences/Cancer Hospital Affiliated to Shanxi Medical University Taiyuan Shanxi China

**Keywords:** adjuvant therapy, esophageal squamous cell carcinoma, neoadjuvant therapy, survival

## Abstract

Controversies persist regarding the optimal regimens of neoadjuvant and adjuvant therapies for locally advanced esophageal squamous cell carcinoma (ESCC). This real‐world cohort study aimed to compare the efficacy of neoadjuvant chemotherapy (NCT), chemoradiotherapy (NCRT), and chemoimmunotherapy (NCIT), and to clarify the survival contributions of adjuvant strategies. We retrospectively enrolled 1818 ESCC patients across four high‐volume Chinese medical centers. After propensity score matching, pathological tumor response and tumor downstaging followed the hierarchy NCRT > NCIT > NCT, yet no significant differences were detected in overall survival (OS) or progression‐free survival (PFS). Subgroup analyses revealed superior OS benefits of NCIT in never‑smokers, nondrinkers, and patients with clinical N2–3 disease compared with NCT. While NCRT showed greater PFS benefit than NCIT as clinical T stage advanced, its OS advantage was restricted to obese individuals. Adjuvant immunotherapy extended PFS for patients receiving prior NCIT, whereas adjuvant chemotherapy delivered survival benefits to those with pathological lymph node metastasis. We constructed optimal treatment rule (OTR) decision trees with moderate‐to‐good predictive capacity to guide selection of neoadjuvant and adjuvant therapies based on survival benefit. These results highlight the necessity of precision perioperative therapy for ESCC. The established OTR decision trees can support individualized clinical decision‐making.

## Graphical abstract

Optimal treatment rule (OTR) decision trees for selecting neoadjuvant therapies based on overall survival, using core subgroup variables as predictors. The three values in each box represent, from left to right, the probability of benefit from neoadjuvant chemotherapy (NCT), neoadjuvant chemoimmunotherapy (NCIT), and neoadjuvant chemoradiotherapy (NCRT), respectively.

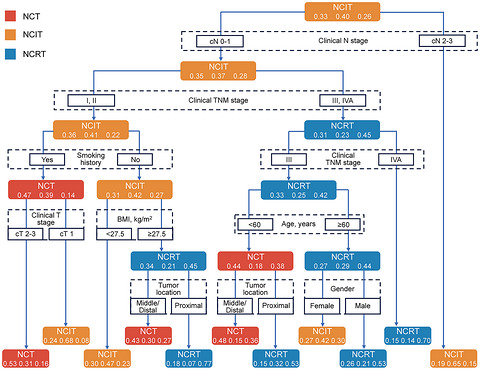

## 1 Introduction

Esophageal cancer ranks as the 11th most commonly malignancy and the seventh leading cause of cancer‐related mortality globally [1]. China accounts for nearly 40% of global cases and deaths, with squamous cell carcinoma (ESCC) as the predominant histology [1, 2]. Due to the lack of early symptoms and sensitive biomarkers, most ESCC cases are diagnosed at advanced stages. For locally advanced ESCC, neoadjuvant therapy followed by surgery, with subsequent adjuvant therapy when indicated, represent the standard of care [3, 4]; however, a precision approach to neoadjuvant and subsequent adjuvant strategy has not yet been established.

Neoadjuvant chemotherapy (NCT), chemoradiotherapy (NCRT), or chemoimmunotherapy (NCIT) can downstage tumors and improve resectability [5, 6]. Nevertheless, the optimal neoadjuvant strategy for ESCC patients remains controversial. While NCRT is widely used for esophageal adenocarcinomas in Western countries [4], NCT is commonly adopted in China and has demonstrated noninferior survival compared with NCRT [6]. The introduction of immune checkpoint inhibitors has further complicated therapeutic decision‐making. Existing observational studies do not conclusively demonstrate a survival advantage of NCIT over NCT [7, 8]. The ESCORT‐NEO/NCCES01 trial, the first Phase 3 study comparing NCIT with NCT in esophageal cancers, showed significantly improved complete pathological response (CPR) and major pathological response (MPR) rates with NCIT and comparable treatment‐related adverse events [5]. The HCHTOG1909 study confirmed these elevated pathological response rates [9]. However, survival data from these clinical trials are still maturing. Notably, large‐scale real‐world comparisons of NCT, NCIT, and NCRT are lacking, and the populations most likely to benefit from each strategy require clarification.

The role of adjuvant therapy after neoadjuvant treatment and surgery has been underexplored in ESCC. Some studies suggest short‐term survival benefit for ESCC patients receiving adjuvant therapy after neoadjuvant treatment and radical surgery, without long‐term survival benefit [10, 11]. Postoperative immunotherapy offers new adjuvant options, and adjuvant nivolumab has shown promise for improving survival in ESCC patients undergoing NCRT followed by surgery [12]. In practice, adjuvant regimens vary and may include chemotherapy, radiotherapy, immunotherapy, or combinations thereof. The efficacy of these approaches and their optimal selection based on preceding neoadjuvant therapies have not been systematically evaluated in ESCC.

This multicenter real‐world study aimed to inform precision neoadjuvant and subsequent adjuvant therapies for ESCC patients. We compared the efficacy of NCT, NCIT, and NCRT, identified subgroups that benefit from each approach, and evaluated the role and selection of adjuvant therapy following neoadjuvant treatment and surgery. Additionally, we established optimal treatment rule (OTR) decision trees to facilitate therapy selection based on survival benefit.

## 2 Results

### 2.1 Patient Recruitment

**FIGURE 1:**
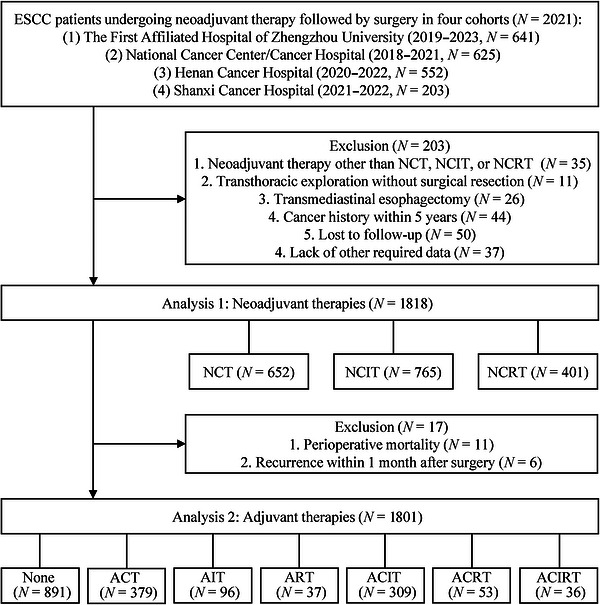
Participant enrollment flowchart. ACT, adjuvant chemotherapy; ACIRT, adjuvant chemo‐immuno‐radiotherapy; ACIT, adjuvant chemoimmunotherapy; ACRT, adjuvant chemoradiotherapy; AIT, adjuvant immunotherapy; ART, adjuvant radiotherapy; ESCC, esophageal squamous cell carcinoma; NCIT, neoadjuvant chemoimmunotherapy; NCRT, neoadjuvant chemoradiotherapy; NCT, neoadjuvant chemotherapy.

During the study period, 2021 ESCC patients undergoing neoadjuvant therapy followed by surgery were assessed for inclusion (Figure 1). After excluding 203 patients with reasons, 1818 patients were included in the neoadjuvant therapy analysis. Following exclusion of perioperative mortality (11 cases) and early recurrence (six cases), 1801 patients were included in the adjuvant therapy analysis.

### 2.2 Analysis of Neoadjuvant Therapy

#### 2.2.1 Groups and Characteristics

The NCT, NCIT, and NCRT groups consisted of 652 (35.9%), 765 (42.1%), and 401 (22.1%) ESCC patients, respectively (Table 1). Patients in the NCRT group were younger, had a higher proportion of males, and a higher prevalence of alcohol misuse compared with the NCT and NCIT groups. The NCRT group had more advanced clinical T stage and clinical tumor–node–metastasis (TNM) stage than the NCT and NCIT groups, while the NCIT group had a more advanced clinical N stage than the other two groups. Propensity score matching (PSM) was subsequently conducted (Table S1), the three groups were matched in pairwise combinations: NCT versus NCIT (1:1, 576 pairs), NCT versus NCRT (1:1, 393 pairs), and NCIT versus NCRT (2:1, 592 NCIT and 379 NCRT patients). No significant differences in clinical, surgical, or tumor characteristics were observed among the matched pairs.

**TABLE 1:** Baseline characteristics by neoadjuvant therapy.

Characteristics	Neoadjuvant therapies			P‐total	P1	P2	P3
	NCT (*N* = 652)	NCIT (*N* = 765)	NCRT (*N* = 401)				
Demographics							
Age, year	64.2 ± 8.0	63.8 ± 7.7	62.7 ± 8.0	0.011	0.30	0.003	0.029
Gender							
Male	491 (75.3)	577 (75.4)	325 (81.0)	0.060	0.96	0.030	0.029
Female	161 (24.7)	188 (24.6)	76 (19.0)				
Smoking history							
Yes	365 (56.0)	399 (52.2)	229 (57.1)	0.19	0.15	0.72	0.11
No	287 (44.0)	366 (47.8)	172 (42.9)				
Alcohol misuse							
Yes	288 (44.2)	323 (42.2)	211 (52.6)	0.003	0.46	0.008	0.001
No	364 (55.8)	442 (57.8)	190 (47.4)				
Body mass index, kg/m^2^	23.6 ± 3.2	23.3 ± 3.3	23.4 ± 3.2	0.12	0.041	0.21	0.62
Comorbidities							
Cardiovascular disease							
Yes	89 (13.7)	93 (12.2)	47 (11.7)	0.59	0.40	0.37	0.83
No	563 (86.3)	672 (87.8)	354 (88.3)				
Pulmonary disease							
Yes	22 (3.4)	19 (2.5)	17 (4.2)	0.25	0.32	0.47	0.10
No	630 (96.6)	746 (97.5)	384 (95.8)				
Diabetes							
Yes	69 (10.6)	73 (9.5)	45 (11.2)	0.64	0.52	0.75	0.37
No	583 (89.4)	692 (90.5)	356 (88.8)				
Charlson comorbidity index							
<3	472 (72.4)	584 (76.3)	294 (73.3)	0.21	0.089	0.74	0.26
≥3	180 (27.6)	181 (23.7)	107 (26.7)				
Pulmonary function							
FEV1.0, L	2.59 (2.13–3.04)	2.70 (2.19–3.09)	2.62 (2.25–3.18)	0.27	0.12	0.25	0.93
DLCO‐SB, mmol/min/kPa	6.91 (2.89–8.03)	6.93 (5.90–8.07)	7.13 (5.99–8.20)	0.23	0.92	0.11	0.14
Surgical parameters							
Surgery approach							
McKeown	597 (91.6)	742 (97.0)	392 (97.8)	<0.001	<0.001	<0.001	0.58
Ivor Lewis	40 (6.1)	16 (2.1)	5 (1.2)				
Sweet	15 (2.3)	7 (0.9)	4 (1.0)				
Surgery technique							
VATS	577 (88.5)	719 (94)	364 (90.8)	<0.001	<0.001	0.007	0.006
Robot‐assisted surgery	14 (2.1)	33 (4.3)	17 (4.2)				
Thoracotomy	61 (9.4)	13 (1.7)	20 (5.0)				
Tumor characteristics							
Tumor location							
Proximal third	81 (12.5)	106 (13.9)	80 (20.5)	0.35	0.39	0.45	0.16
Middle third	320 (49.5)	380 (49.9)	162 (41.4)				
Distal third	245 (37.9)	275 (36.1)	149 (38.1)				
Clinical T stage							
cT1	72 (11.0)	139 (18.2)	50 (12.5)	<0.001	0.058	0.001	<0.001
cT2	121 (18.6)	105 (13.7)	41 (10.2)				
cT3	439 (67.3)	506 (66.1)	274 (68.3)				
cT4	20 (3.1)	15 (2.0)	36 (9.0)				
Clinical N stage							
cN0	283 (43.4)	276 (36.1)	153 (38.2)	<0.001	<0.001	0.66	<0.001
cN1	255 (39.1)	282 (36.9)	195 (48.6)				
cN2	93 (14.3)	178 (23.3)	53 (13.2)				
cN3	21 (3.2)	29 (3.8)	0				
Clinical TNM stage							
I	66 (10.1)	113 (14.8)	41 (10.2)	0.010	0.97	0.005	0.006
II	300 (46)	289 (37.8)	145 (36.2)				
III	247 (37.9)	322 (42.1)	179 (44.6)				
IV	39 (6.0)	41 (5.4)	36 (9.0)				

Data are presented as mean ± standard deviation, number (percentage), or median (interquartile range).

Group differences were assessed using ANOVA, Pearson's chi‐squared test, Fisher's exact test, Mann–Whitney *U*‐test, or Kruskal–Wallis test. *p* Values are denoted as follows: P‐total (comparison among NCT, NCIT, and NCRT groups), P1 (NCIT vs. NCT), P2 (NCRT vs. NCT), and P3 (NCIT vs. NCRT).

*Abbreviations*: DLCO‐SB, single‐breath diffusing capacity of the lung for carbon monoxide; FEV1, forced expiratory volume in 1 s; NCIT, neoadjuvant chemoimmunotherapy; NCRT, neoadjuvant chemoradiotherapy; NCT, neoadjuvant chemotherapy; VATS, video‐assisted thoracic surgery.

#### 2.2.2 Perioperative Outcomes

During surgical resection, the three groups showed no significant difference in operation time or estimated blood loss. However, the number of dissected lymph nodes was highest in the NCIT group, followed by the NCT group, and lowest in the NCRT group (Table S2). After PSM, the difference in lymph node yield persisted, while no significant difference in surgery radicality was observed (Table 2). Following surgery, the groups showed no significant differences in overall complication rates, complication severity, or postoperative hospital stay, both before and after PSM.

**TABLE 2:** Short‐term outcomes by neoadjuvant therapy after propensity score matching.

Characteristics	NCT vs. NCIT (ratio 1:1)			NCT vs. NCRT (ratio 1:1)			NCIT vs. NCRT (ratio 2:1)		
	NCT (*N* = 576)	NCIT (*N* = 576)	*p* Value	NCT (*N* = 393)	NCRT (*N* = 393)	*p* Value	NCIT (*N* = 592)	NCRT (*N* = 379)	*p* Value
Intraoperative outcomes									
Operation time, min	285 (243–335)	284 (235–340)	0.59	280 (243–330)	280 (235–326)	0.16	283(235–339)	280 (235–327)	0.25
Estimated blood loss, mL	100 (50–200)	100 (50–200)	0.13	100 (50–200)	100 (50–200)	0.43	100 (80–200)	100 (50–200)	0.27
Lymph node dissection, no.	27 (20–35)	30 (23–40)	<0.001	27 (19–35)	24 (17–33)	0.002	30 (22–40)	24 (17–33)	<0.001
Radicality of surgery									
R0	574 (99.7)	571 (99.1)	0.45	391 (99.5)	387 (98.0)	0.29	587 (99.2)	373 (97.9)	0.45
R1	2 (0.3)	5 (0.9)		2 (0.5)	6 (2.0)		5 (0.8)	6 (2.1)	
Postoperative complications									
Specific complications									
Pneumonia	92 (16.0)	88 (15.3)	0.75	58 (14.8)	56 (14.2)	0.84	90 (15.2)	51 (13.5)	0.45
Pleural effusion	67 (11.6)	87 (15.1)	0.083	48 (12.2)	60 (15.3)	0.21	96 (16.2)	53 (14.0)	0.35
Pneumothorax	6 (1.0)	12 (2.1)	0.15	4 (1.0)	6 (1.5)	0.75	13 (2.2)	6 (1.6)	0.50
Respiratory failure	8 (1.4)	9 (1.6)	0.81	7 (1.8)	1 (0.3)	0.076	8 (1.4)	1 (0.3)	0.17
Myocardial arrhythmia	5 (0.9)	11 (1.9)	0.13	1 (0.3)	6 (1.5)	0.13	11 (1.9)	6 (1.6)	0.75
Congestive heart failure	1 (0.2)	3 (0.5)	0.62	2 (0.5)	3 (0.8)	1.00	3 (0.5)	3 (0.8)	0.89
Anastomotic leak	30 (5.2)	44 (7.6)	0.092	20 (5.1)	30 (7.6)	0.14	41 (6.9)	30 (7.9)	0.56
Wound infection	9 (1.6)	8 (1.4)	0.81	2 (0.5)	5 (1.3)	0.45	8 (1.4)	5 (1.3)	0.97
Chylothorax	1 (0.2)	3 (0.5)	0.62	1 (0.3)	0	1.00	2 (0.3)	0	0.52
Overall complications									
Yes	138 (24.0)	157 (27.3)	0.20	89 (22.6)	94 (23.9)	0.67	160 (27.0)	87 (23.0)	0.16
No	438 (76.0)	419 (72.7)		304 (77.4)	299 (76.1)		432 (73.0)	292 (77.0)	
Clavien–Dindo complication grades									
I	10 (1.7)	10 (1.7)	0.17	7 (1.8)	7 (1.8)	0.70	13 (2.2)	7 (1.8)	0.16
II	64 (11.1)	66 (11.5)		39 (9.9)	37 (9.4)		62 (10.5)	32 (8.4)	
IIIA	46 (8.0)	63 (10.9)		28 (7.1)	46 (11.7)		70 (11.8)	44 (11.6)	
IIIB	5 (0.9)	6 (1.0)		3 (0.8)	0		5 (0.8)	0	
IV	11 (1.9)	11 (1.9)		9 (2.3)	2 (0.5)		9 (1.5)	2 (0.5)	
V	2 (0.3)	1 (0.2)		3 (0.8)	2 (0.5)		1 (0.2)	2 (0.5)	
Postoperative hospital stay, days	9 (8–12)	10 (8–13)	0.17	9 (8–12)	10 (8–12)	0.73	10 (8–13)	10 (8–12)	0.24
Pathological outcomes									
Complete pathologic response									
Yes	58 (10.1)	147 (25.5)	<0.001	36 (9.2)	136 (34.6)	<0.001	159 (26.9)	129 (34.0)	0.017
No	518 (89.9)	429 (74.5)		357 (90.8)	257 (65.4)		433 (73.1)	250 (66.0)	
Major pathological response									
Yes	129 (22.4)	251 (43.6)	<0.001	85 (21.6)	259 (65.9)	<0.001	271 (45.8)	253 (66.8)	<0.001
No	447 (77.6)	325 (56.4)		308 (78.4)	134 (34.1)		321 (54.2)	126 (33.2)	
ypT stage									
ypT0	78 (13.5)	168 (29.2)	<0.001	51 (13.0)	172 (43.8)	<0.001	183 (30.9)	164 (43.3)	0.002
ypT1	103 (17.9)	94 (16.3)		66 (16.8)	56 (14.2)		108 (18.2)	58 (15.3)	
ypT2	155 (26.9)	157 (27.3)		101 (25.7)	74 (18.8)		154 (26.0)	72 (19.0)	
ypT3	234 (40.6)	151 (26.2)		168 (42.7)	87 (22.1)		144 (24.3)	81 (21.4)	
ypT4	6 (1.1)	6 (1.0)		7 (1.8)	4 (1.0)		3 (0.5)	4 (1.1)	
ypN stage									
ypN0	309 (53.6)	360 (62.5)	0.002	190 (48.3)	278 (70.7)	<0.001	372 (62.8)	266 (70.2)	0.014
ypN1	160 (27.8)	139 (24.1)		121 (30.8)	79 (20.1)		146 (24.7)	79 (20.8)	
ypN2	82 (14.2)	53 (9.2)		64 (16.3)	30 (7.6)		54 (9.1)	28 (7.4)	
ypN3	25 (4.3)	24 (4.2)		18 (4.6)	6 (1.5)		20 (3.4)	6 (1.6)	
ypTNM stage									
ypI	216 (37.5)	397 (68.9)	<0.001	130 (33.1)	230 (58.5)	<0.001	312 (52.7)	222 (58.6)	0.026
ypII	92 (16.0)	60 (10.4)		58 (14.8)	46 (11.7)		57 (9.6)	41 (10.8)	
ypIIIA	81 (14.1)	84 (14.6)		57 (14.5)	52 (13.2)		89 (15.0)	52 (13.7)	
ypIIIB	157 (27.3)	108 (18.8)		125 (31.8)	57 (14.5)		114 (19.3)	56 (14.8)	
ypIV	30 (5.2)	27 (4.7)		23 (5.9)	8 (2.0)		20 (3.4)	8 (2.1)	
Lymphovascular invasion									
Yes	175 (30.4)	114 (19.8)	<0.001	124 (31.6)	49 (12.5)	<0.001	109 (18.4)	44 (11.6)	0.005
No	401 (69.6)	462 (80.2)		269 (68.4)	344 (87.5)		483 (81.6)	335 (88.4)	
Perineural invasion									
Yes	120 (20.8)	82 (14.2)	0.003	89 (22.6)	45 (11.5)	<0.001	71 (12.0)	39 (10.3)	0.41
No	456 (79.2)	494 (85.8)		304 (77.4)	348 (88.5)		521 (88.0)	340 (89.7)	
Adjuvant therapy									
Yes	325 (56.9)	304 (53.1)	0.19	237 (61.2)	137 (35.2)	<0.001	321 (54.6)	129 (34.4)	<0.001
No	246 (43.1)	269 (46.9)		150 (38.8)	252 (64.8)		267 (45.4)	246 (65.6)	

Data are presented as number (percentage) or median (interquartile range).

Group differences were assessed using Pearson's chi‐squared test, Fisher's exact test, or Mann–Whitney *U*‐test.

*Abbreviations*: NCIT, neoadjuvant chemoimmunotherapy; NCRT, neoadjuvant chemoradiotherapy; NCT, neoadjuvant chemotherapy.

#### 2.2.3 Pathological Results

The NCRT group achieved the highest CPR and MPR rates, followed by the NCIT group, with the lowest rates in the NCT group (Table S2). These differences remained after PSM (Table 2). The NCRT group showed the most favorable pathological downstaging (ypT, ypN, and ypTNM), followed by the NCIT group, with the least downstaging observed in the NCT group, both before and after PSM. Correspondingly, the lymphovascular invasion positivity was lowest in the NCRT group, followed by the NCIT group, and highest in the NCT group, both before and after PSM. Perineural invasion rates were comparable between the NCRT and NCIT groups, both of which were lower than that in the NCT group.

#### 2.2.4 Recurrence Patterns

The three groups showed no significant differences in overall recurrence, locoregional recurrence, distant metastasis, or combined local and distant recurrence, both before and after PSM (Tables S3 and S4). Notably, the NCRT group showed trends toward decreased locoregional recurrence compared with both NCT and NCIT after PSM (NCRT vs. NCT: 7.9 vs. 10.9%, *p* = 0.14; NCRT vs. NCIT: 6.9 vs. 9.8%, *p* = 0.11). The incidence of supraclavicular lymph node metastasis in the NCRT group was nonsignificantly lower than in the NCT group (2.8 vs. 5.1%, *p* = 0.099).

#### 2.2.5 Survival Outcomes

##### 2.2.5.1 Univariable Analysis

**FIGURE 2:**
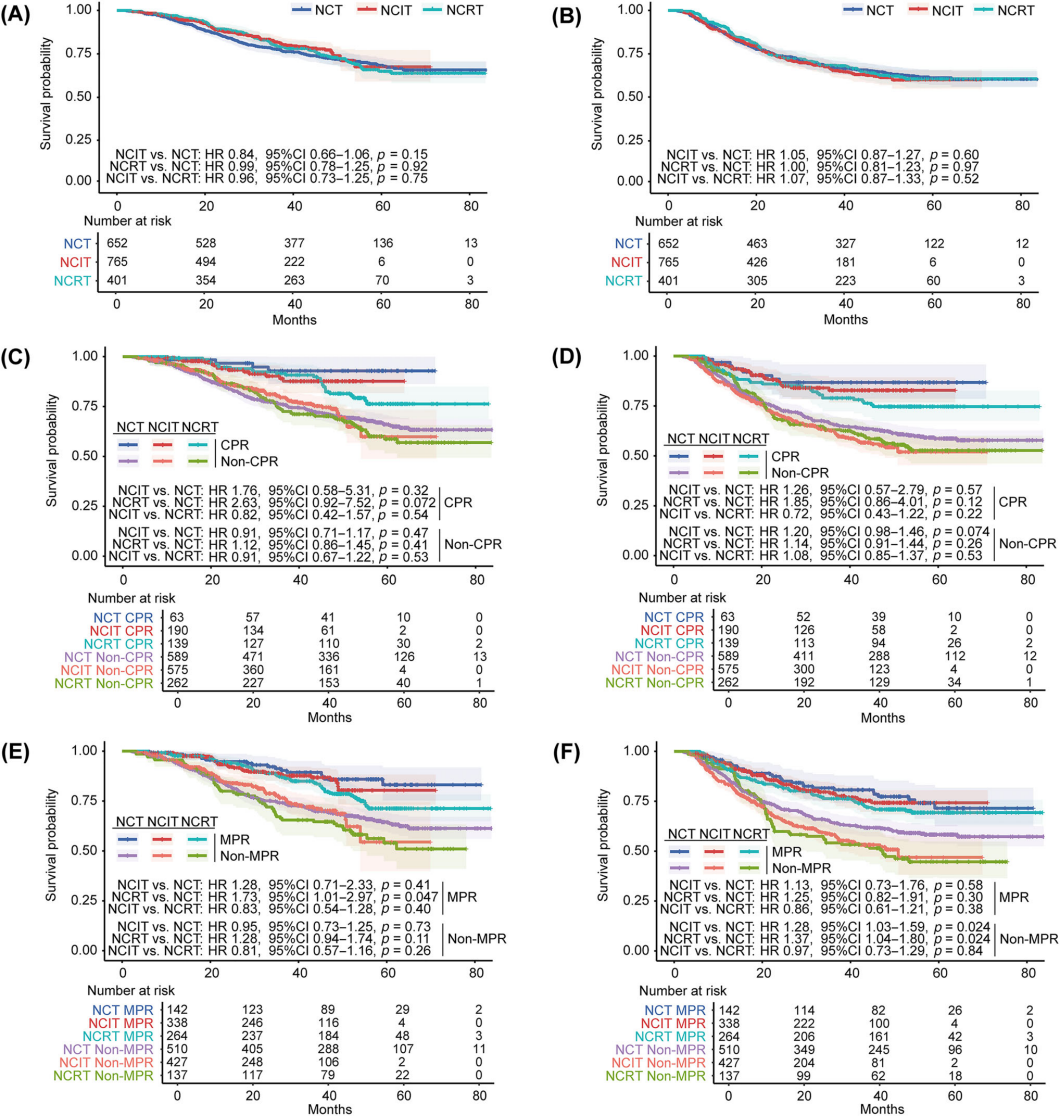
Overall survival (OS) and progression‐free survival (PFS) by neoadjuvant therapy. (A and B) OS (A) and PFS (B) comparing NCT, NCIT, or NCRT before propensity score matching. (C and D) OS (C) and PFS (D) for patients receiving NCT, NCIT, or NCRT, stratified by CPR status. (E and F) OS (E) and PFS (F) for patients receiving NCT, NCIT, or NCRT, stratified by MPR status. CI, confidence interval; CPR, complete pathologic response; HR, hazard ratio; MPR, major pathological response; NCIT, neoadjuvant chemoimmunotherapy; NCRT, neoadjuvant chemoradiotherapy; NCT, neoadjuvant chemotherapy.

The median follow‐up for survivors was 43.9 months (interquartile range: 23.0–54.5). The three groups showed no significant difference in overall survival (OS) or progression‐free survival (PFS), both before and after PSM (Figures 2A,B and S1A–F). A trend toward better OS with NCIT versus NCT observed before PSM diminished after matching.

##### 2.2.5.2 Survival Analysis by Tumor Response

Patients achieving CPR and MPR after neoadjuvant therapy had significantly better OS and PFS than those who did not (Figure S2A–D). Among responders (CPR or MPR), those receiving NCT showed trends toward better OS and PFS than those receiving NCRT, while NCIT showing intermediate benefit (Figure 2C–F). Among nonresponders (non‐CPR or non‐MPR), those receiving NCT showed trends toward better PFS than those receiving NCIT or NCRT (Figure 2D,F). The OS benefit of non‐MPR patients receiving NCRT was numerically worse than that of those receiving NCT, although the difference was not significant (Figure 2E).

##### 2.2.5.3 Survival Analysis According to Adjuvant Therapy

Among patients receiving adjuvant therapy, those who had undergone NCRT showed significantly worse OS and PFS than those who had undergone NCT or NCIT (Figure S2E,F). Conversely, among patients who did not receive adjuvant therapy, those who had undergone NCRT showed trends toward better OS and PFS than those who had undergone NCT or NCIT (Figure S2E,F).

##### 2.2.5.4 Comprehensive Subgroup Analysis

**FIGURE 3:**
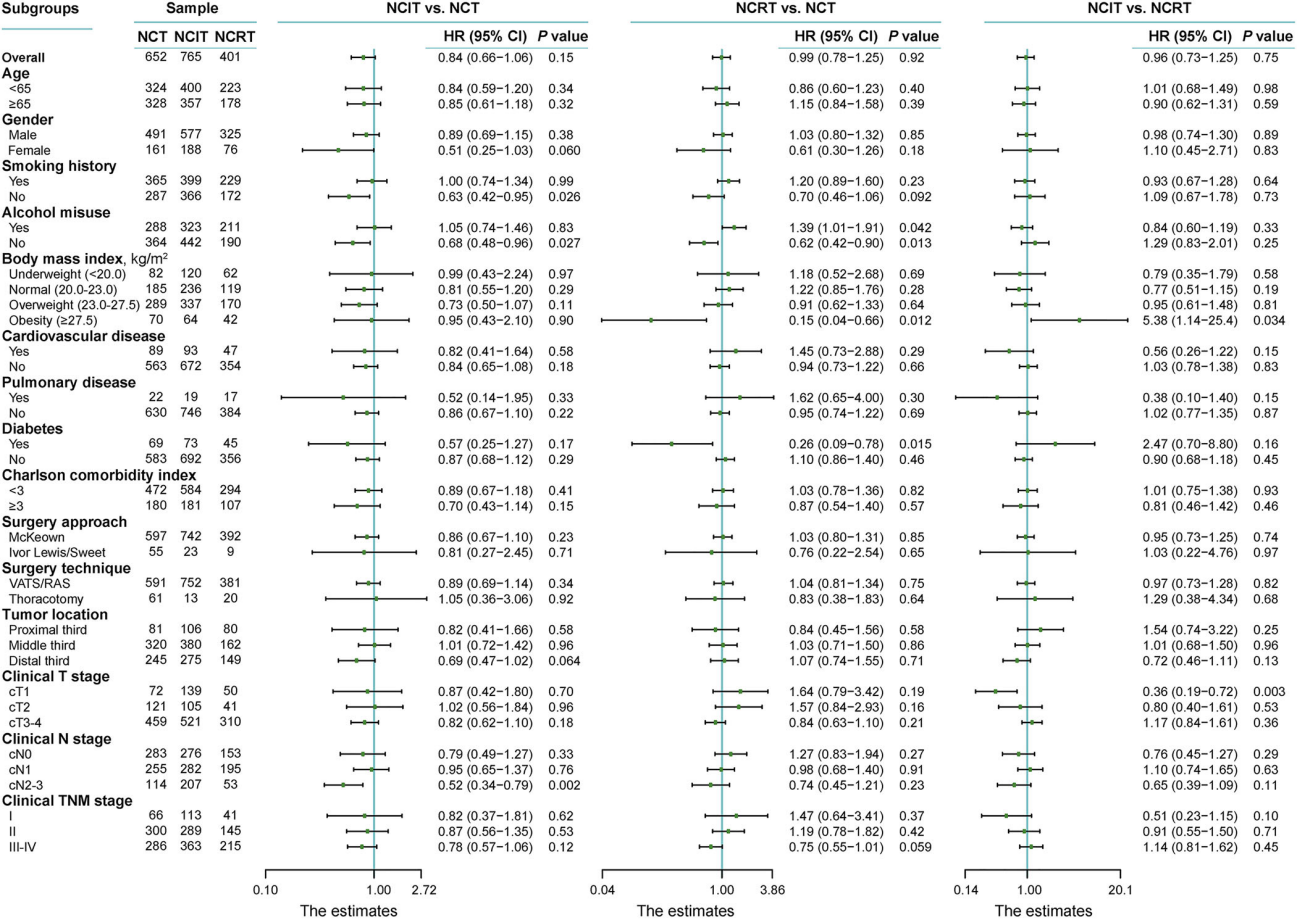
Subgroup analysis of overall survival by neoadjuvant therapy. Forest plot showing hazard ratios for overall survival among patients treated with different neoadjuvant therapies followed by surgery, across various patient subgroups. CI, confidence interval; HR, hazard ratio; NCIT, neoadjuvant chemoimmunotherapy; NCRT, neoadjuvant chemoradiotherapy; NCT, neoadjuvant chemotherapy; RAT, robot‐assisted surgery; VATS, video‐assisted thoracic surgery.

Based on subgroup analysis (Figures 3 and S3), among patients with no history of smoking or alcohol misuse, the NCIT and NCRT groups showed superior OS to the NCT group. The NCRT group further showed trends toward greater PFS in these populations. In contrast, for patients with alcohol misuse, the NCRT group showed poorer OS and PFS than the NCT group, and the NCIT group also showed trends toward poorer PFS. Notably, the NCRT group showed superior OS to the NCT and NCIT groups in obese patients or those with diabetes. PFS after NCRT was also nonsignificantly longer than after NCT or NCIT in these populations. The NCIT group showed trends toward greater OS than the NCT and NCRT groups in patients with ESCC in the distal third of the esophagus. With advancing clinical T stage, N stage, and TNM stage, the OS and PFS benefit of NCIT and NCRT over NCT increased. For ESCC of clinical T3–4 or TNM III–IV, both NCIT and NCRT showed trends toward better OS than NCT, while the NCRT group showed trends toward better PFS than NCIT. In contrast, for clinical T1 stage ESCC, the NCRT group showed poorer OS and PFS. Additionally, the NCIT group showed better OS than both NCT and NCRT groups in ESCC with clinical N2–3 stages, although this superiority was only retained in PFS when compared with NCT.

#### 2.2.6 OTR Decision Trees for OS and PFS Regarding Neoadjuvant Therapy

**FIGURE 4:**
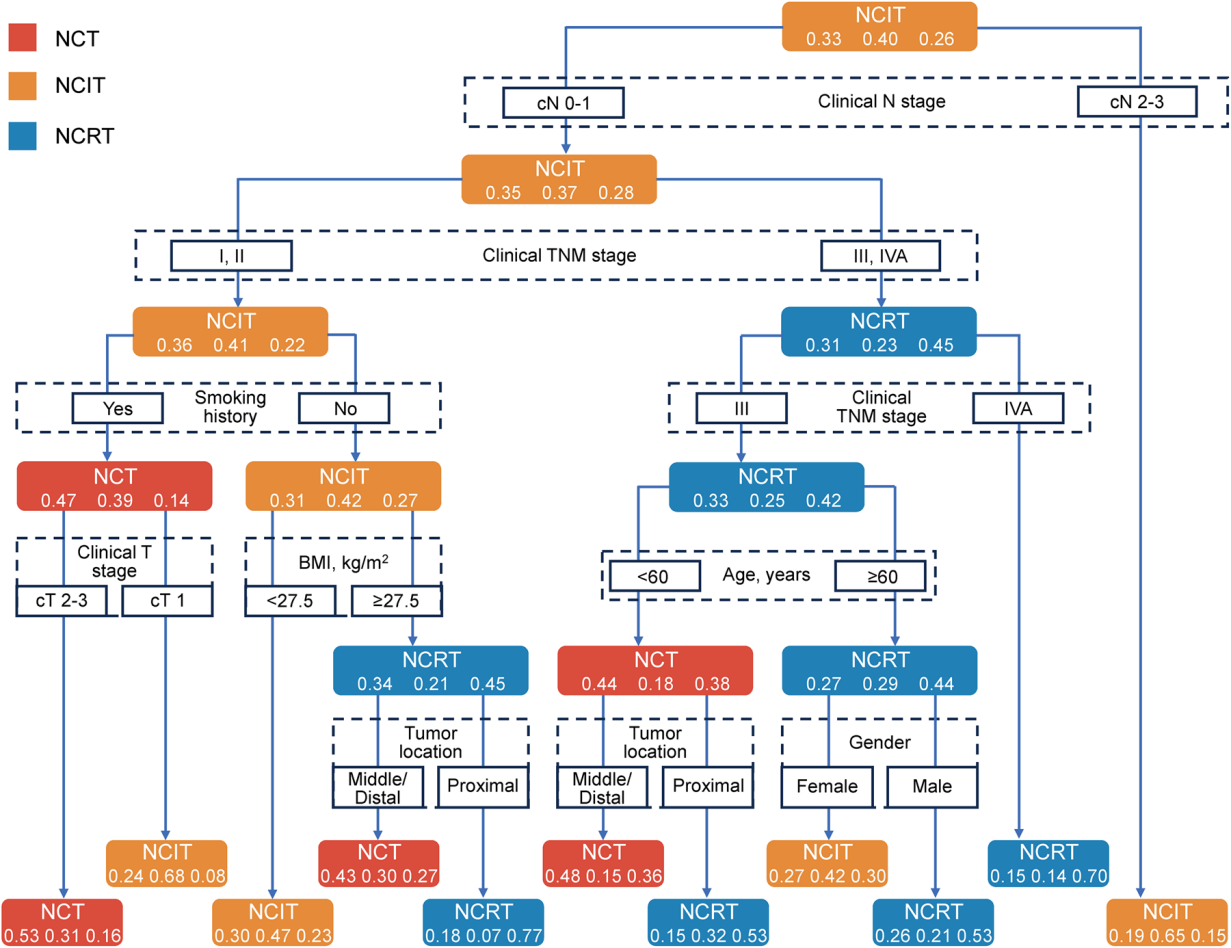
Optimal treatment rule (OTR) decision trees for selecting neoadjuvant therapies based on overall survival, using core subgroup variables as predictors. The three values in each box represent, from left to right, the probability of benefit from neoadjuvant chemotherapy (NCT), neoadjuvant chemoimmunotherapy (NCIT), and neoadjuvant chemoradiotherapy (NCRT). BMI, body mass index; NCIT, neoadjuvant chemoimmunotherapy; NCRT, neoadjuvant chemoradiotherapy; NCT, neoadjuvant chemotherapy.

OTR decision trees were constructed to translate the aforementioned survival analysis results into clinical decision‐making for selecting neoadjuvant therapies in ESCC patients. The core subgroup variables measured before neoadjuvant therapy were included as candidate predictors. As illustrated in Figures 4 and S4, the OTR decision trees for OS and PFS consistently incorporated parameters such as age, gender, smoking history, body mass index (BMI), tumor location, and clinical T, N, and TNM stages, albeit with different decision priorities. Notably, clinical N and TNM stages consistently ranked as the top two predictors. The included parameters were generally consistent with the core subgroup variables reported in Section 2.2.5.4. The selection of neoadjuvant therapies guided by the OTR decision trees also aligned with the results of subgroup analyses. The predictive performance of the OTR decision trees, as measured by the concordance index (C‑index), was 0.682 (95% confidence interval [CI] 0.651–0.730) for OS and 0.658 (95%CI 0.630–0.699) for PFS.

### 2.3 Analysis of Adjuvant Therapy

#### 2.3.1 Groups and Characteristics

Among the 1801 patients included in the adjuvant therapy analysis, 910 (50.5%) received adjuvant therapy. Patients undergoing adjuvant therapy more commonly had undergone NCT and NCIT, had lower CPR and MPR rates, more advanced pathological stages, and higher rates of invasion events (Table S5). PSM was subsequently conducted at a 1:1 ratio. Finally, 695 matched pairs of patients with and without adjuvant therapy were identified, and no significant differences in clinicopathological characteristics were observed after PSM (Table S5).

#### 2.3.2 Survival Outcomes According to Adjuvant Therapy

**FIGURE 5:**
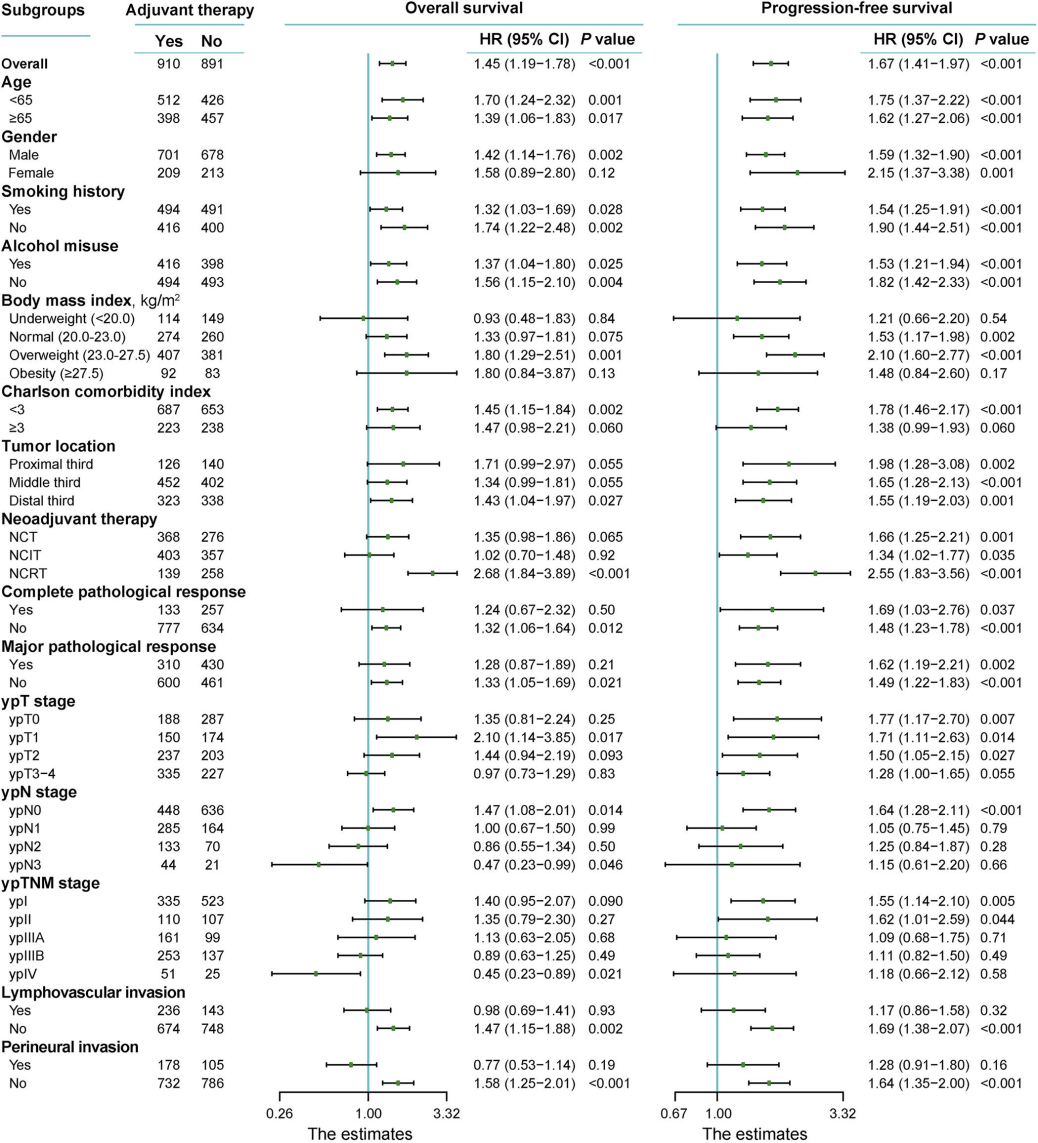
Subgroup analysis of the impact of adjuvant therapy on survival. Forest plot showing hazard ratios for overall survival and progression‐free survival with versus without adjuvant therapy following neoadjuvant therapy and surgery, across various patient subgroups. CI, confidence interval; HR, hazard ratio.

Patients undergoing adjuvant therapy showed compromised OS compared with those without adjuvant therapy, both before and after PSM (Figure S5A,B; before PSM: hazard ratio [HR] 1.45, 95%CI 1.19–1.78; after PSM: HR 1.34, 95%CI 1.06–1.69). Adjuvant therapy appeared to reduce mortality for approximately 20 months after surgery but was associated with increased mortality thereafter. The PFS of patients undergoing adjuvant therapy was consistently poorer than that of others (Figure S5C,D; before PSM: HR 1.67, 95%CI 1.41–1.97; after PSM: HR 1.49, 95%CI 1.22–1.81). Subgroup analysis showed that adjuvant therapy was associated with compromised OS and PFS in most populations stratified by clinical, surgical, and pathological characteristics (Figure 5). No significant survival benefit was observed in patients undergoing adjuvant therapy regardless of the CPR or MPR status. The potential benefit of adjuvant therapy on OS and PFS increased with advancing pathological T, N, and TNM stages. Specifically, adjuvant therapy improved OS in ESCC with ypN3 or ypTNM IV stages.

#### 2.3.3 Survival Outcomes of Specific Adjuvant Strategies

Among patients undergoing adjuvant therapy, the regimens included chemotherapy (ACT), immunotherapy (AIT), radiotherapy (ART), chemoimmunotherapy (ACIT), chemoradiotherapy (ACRT), and chemo‐immuno‐radiotherapy (ACIRT). Overall, no adjuvant strategy improved OS or PFS; ART, ACIT, ACRT, and ACIRT showed adverse effects (Figure S6A,B). Among patients who had undergone NCIT, AIT improved PFS (HR 0.52, 95%CI 0.27–0.99), with nonsignificantly improved OS, while no adjuvant strategy improved survival for patients who had undergone NCT or NCRT (Figure S6C–H). Based on subgroup analysis of pathological characteristics (Figures S6I–T and S7A–P), apart from ACT, other adjuvant strategies rarely improved survival. ACT improved OS and PFS in ESCC with ypN1–3 (Figure S6O,P; OS: HR 0.65, 95%CI 0.46–0.92; PFS: HR 0.78, 95%CI 0.61–0.99) or ypTNM III–IV (Figure S6S,T; OS: HR 0.65, 95%CI 0.46–0.91; PFS: HR 0.74, 95%CI 0.55–0.99) stages and showed trends toward improving these outcomes in ESCC with lymphovascular invasion (Figure S7K,L). It was also associated with improved OS in patients with perineural invasion (Figure S7O, HR 0.53, 95%CI 0.32–0.87). However, no adjuvant therapies succeeded in improving survival outcomes in subgroups classified based on CPR or MPR status after neoadjuvant therapy (Figure S7A–H).

#### 2.3.4 OTR Decision Trees for OS and PFS Regarding Adjuvant Therapy

**FIGURE 6:**
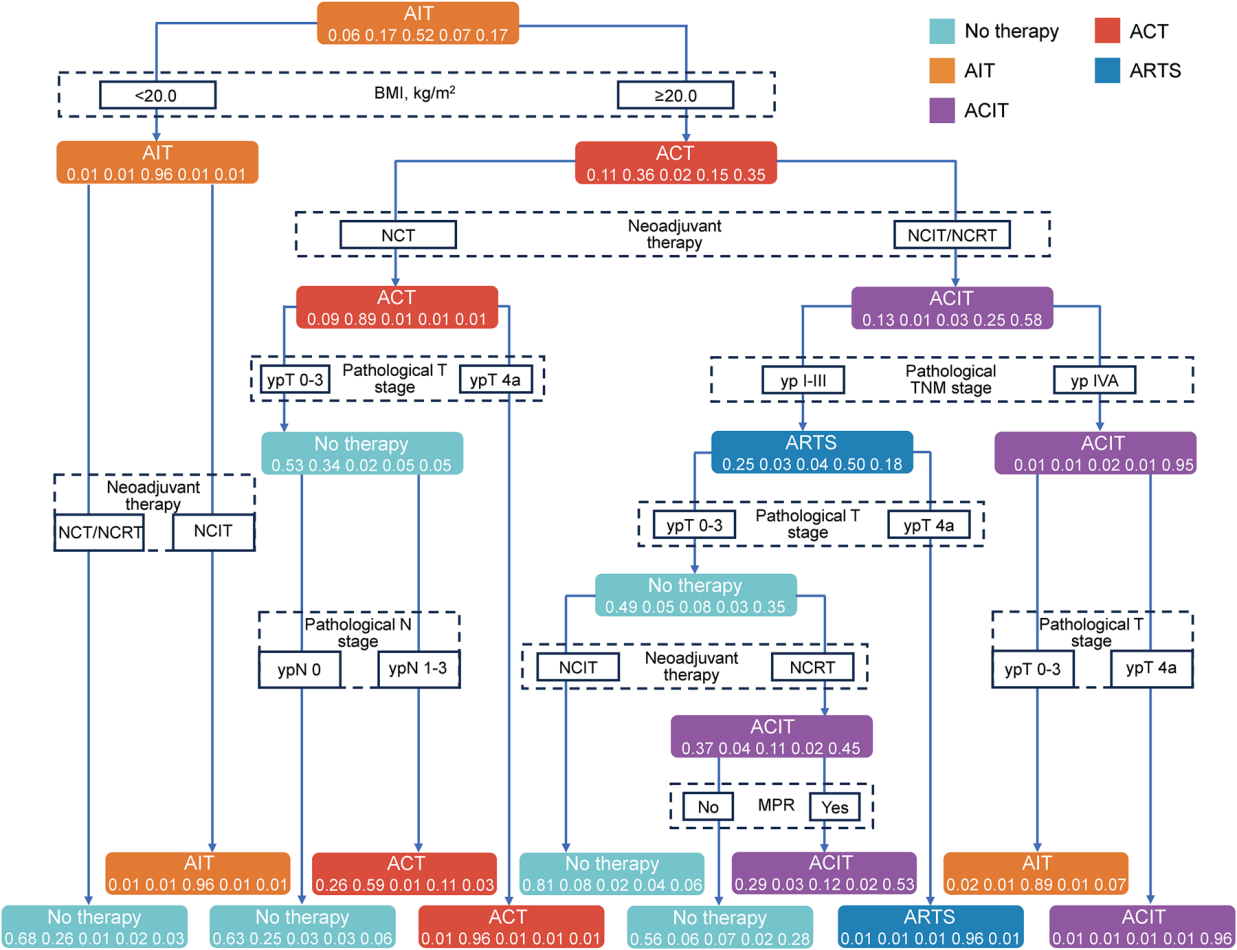
Optimal treatment rule (OTR) decision trees for selecting adjuvant therapies based on overall survival, using core subgroup variables as predictors. The five values in each box represent, from left to right, the probability of benefit from no adjuvant therapy, adjuvant chemotherapy (ACT), adjuvant immunotherapy (AIT), adjuvant radiotherapies (ARTS), and adjuvant chemoimmunotherapy (ACIT). ARTS comprises adjuvant radiotherapy alone, chemoradiotherapy, and chemo‑immuno‑radiotherapy. ACT, adjuvant chemotherapy; ACIT, adjuvant chemoimmunotherapy; AIT, adjuvant immunotherapy; ARTS, adjuvant radiotherapies; BMI, body mass index; MPR, major pathological response; NCIT, neoadjuvant chemoimmunotherapy; NCRT, neoadjuvant chemoradiotherapy; NCT, neoadjuvant chemotherapy.

OTR decision trees were constructed to translate the aforementioned survival analysis results into clinical decision‐making for selecting adjuvant therapies in patients with ESCC. Baseline and pathological parameters were included as candidate predictors. As illustrated in Figure 6, the OTR decision tree for OS included parameters such as BMI, neoadjuvant therapy, MPR, and pathological T, N, and TNM stages, with a C‑index of 0.753 (95%CI 0.722–0.801). In addition to these parameters, the OTR decision tree for PFS further incorporated age, Charlson comorbidity index, and CPR, achieving a C‑index of 0.736 (95%CI 0.708–0.777) (Figure S8). Notably, BMI consistently demonstrated the highest importance in decision‐making for both OS and PFS, followed by neoadjuvant therapy and pathological stages. The included parameters were generally consistent with the core subgroup variables reported in Sections 2.3.2 and 2.3.3. Moreover, the decision to undergo adjuvant therapy and the specific regimens recommended by the OTR decision trees aligned with the results of these subgroup analyses.

## 3 Discussion

This real‐world study systematically investigated the efficacy of neoadjuvant and subsequent adjuvant therapies in ESCC patients, generating novel evidence to guide precision perioperative therapy. Although OS was comparable across groups, NCT, NCIT, and NCRT each conferred survival advantages in distinct patient subsets. Conversely, postoperative adjuvant treatment yielded clinical benefits only within restricted subgroups.

NCT is consistently linked to suboptimal tumor regression, with CPR rates generally below 10% [5, 6]. Although NCRT substantially elevates CPR rates, two pivotal randomized trials—CIMISG1701 [6] and JCOG1109 [13]—failed to demonstrate its significant OS superiority over NCT. NCIT likewise improves CPR to approximately 20–30% among patients with ESCC [5, 14]. An interim analysis of the HCHTOG1909 trial reported an absolute 11.1% improvement in 1‐year OS with NCIT relative to NCT [9], while several retrospective cohorts have failed to demonstrate definitive survival benefit [7, 8]. A recent multicenter study reported superior OS and disease‐free survival with NCIT versus NCRT in ESCC patients, despite comparable CPR rates [15]. Notably, radiotherapy‐containing NCRT enhances locoregional disease control, especially for primary esophageal lesions and regional lymph nodes [16]. This effect partially explains the numerically reduced locoregional recurrence, though no significant difference in distant metastatic risk was observed within 2 years postesophagectomy. Within our real‐world cohort, NCRT produced the highest CPR and MPR rates alongside the most robust tumor downstaging, followed by NCIT, while NCT yielded the weakest pathological regression. Importantly, these superior pathological responses with NCRT did not translate into long‐term survival benefits. Despite improved PFS with NCRT in select subgroups, NCIT demonstrated noninferior OS relative to NCRT and conferred survival advantages over NCT in specific patient populations.

Tumor response to different neoadjuvant therapies may confer differential survival benefits [15]. In our analysis, CPR or MPR achieved with NCT was associated with better OS and PFS than that achieved with NCRT. Conversely, nonresponders to NCT had better PFS than nonresponders to NCIT or NCRT. These phenomena likely arise from a trade‐off between antitumor potency and treatment tolerability. From NCT to NCIT and NCRT, antitumor potency increases, accompanied by greater physiological treatment burden. Both NCIT and NCRT involve more extended and complex treatment protocols than NCT and carry higher risks of adverse events [9, 13]. The greater extent of lymph node dissection observed in the NCIT group in this study—consistent with prior report [8]—also reflects amplified systemic immune and inflammatory activation triggered by immunotherapy. Furthermore, NCRT correlates with more severe sarcopenia and skeletal muscle loss than NCT, a marker of heightened treatment‐induced physiological stress [17]. When the incremental antitumor activity of NCIT or NCRT fails to offset their excess physical toxicity, these regimens cannot deliver meaningful survival improvements over conventional NCT.

This balance between anticancer potency and physical tolerance forms the basis for identifying beneficiary populations and developing precision treatments. Smoking and alcohol misuse drive persistent systemic inflammation and impair treatment tolerability [18, 19]. Both exposures disrupt immune homeostasis and accelerate immunosenescence, particularly blunting T‐cell priming and cytotoxic function [20, 21]; this immune dysfunction substantially attenuates the therapeutic efficacy of immune checkpoint inhibitors. By contrast, obesity—often coupled with diabetes and greater nutritional reserve—confers superior physiological resilience to intensive multimodal therapy [22]. Accordingly, NCIT and NCRT, which demand robust physical tolerance, preferentially benefit ESCC patients without tobacco or alcohol misuse. Similarly, obese individuals with well‐preserved physical tolerance derive greater clinical benefit from NCRT. On the other hand, survival benefit from NCIT and NCRT are more pronounced in ESCC patients with advanced clinical stages, likely attributable to their stronger systemic antitumor activity. To facilitate individualized neoadjuvant treatment decision‐making, we constructed OTR decision tree predictive models. The moderate discriminative performance of these models validates their clinical utility while simultaneously highlighting the limitations of predictions relying solely on baseline clinicopathological variables. Emerging biomarkers, including programmed death‐ligand 1 (PD‐L1) expression [23], tumor microenvironment signatures [24], circulating tumor DNA [25], and multiomics profiles [26], have proven predictive of NCIT efficacy. Integrating these molecular signatures into predictive algorithms may substantially refine clinical decision‐making, an area requiring further prospective validation.

The survival impact of adjuvant therapy following neoadjuvant therapy and curative esophagectomy remains poorly characterized in ESCC. A meta‐analysis by Lee et al. [11] identified a transient 1‐year OS benefit from postoperative adjuvant treatment, which dissipated at the 5‐year landmark. A separate pooled analysis of randomized controlled trials by Wang et al. [10] similarly reported an early 1‐year OS advantage that diminished with extended follow‐up. Consistent with these prior findings, our cohort confirmed a temporary OS benefit within the first 20 months postadjuvant therapy, which reversed into a detrimental long‐term effect; no durable PFS improvement was observed overall. Subgroup stratification revealed that OS gains from adjuvant treatment—particularly ACT—were restricted to patients with advanced pathological stage or high‐risk invasive pathological features. These short‐term benefits likely stem from adjuvant therapy‐mediated suppression of minimal residual disease (MRD), which inhibits early tumor development after esophagectomy [27, 28]. This MRD inhibition effect carries greater clinical weight for patients with advanced pathological disease or aggressive invasive phenotypes, populations with inherently higher MRD prevalence [29]. Nevertheless, these results also underscore the limited capacity of adjuvant regimens to fully eradicate MRD and achieve sustained curative resection; short‐term suppression of residual tumor burden cannot be converted into persistent long‐term survival advantages.

Within our cohort, AIT and ACIT were predominantly administered to patients who received prior NCIT. However, only AIT yielded survival improvements, most notably prolonged PFS. Recent retrospective series corroborate survival benefits of adjuvant immunotherapy among ESCC patients treated with neoadjuvant chemoimmunotherapy, yet the defining beneficial subgroups remain controversial [30, 31, 32]. Xie et al. [30] reported survival gains from AIT exclusively in patients with poor pathological response and advanced pathological stage post‐NCIT, whereas Chen et al. [32] documented opposing results, observing AIT benefits only in patients achieving robust tumor regression after NCIT. Collectively, our data demonstrate that CPR or MPR status after neoadjuvant therapy cannot reliably guide adjuvant treatments. In contrast, advanced pathological stage and high‐risk invasive features—well‐established markers of elevated recurrence risk that predict adjuvant treatment responsiveness [33, 34]—were validated in our cohort as actionable criteria for adjuvant therapy administration, particularly ACT. These complexities highlight the multifaceted nature of adjuvant treatment decision‐making postesophagectomy and necessitate predictive models built on comprehensive multivariable clinicopathological analyses. The OTR decision trees constructed for adjuvant therapy exhibited satisfactory predictive performance for both OS and PFS, supporting their clinical value for risk stratification based on core baseline and postoperative pathological parameters. Baseline BMI ranked as the most impactful predictive variable for both OS and PFS endpoints, followed by neoadjuvant regimen type and pathological tumor stage. Underweight patients are generally discouraged from receiving multimodal adjuvant therapy and may be prioritized for AIT if adjuvant intervention is indicated.

These findings emphasize the critical need to balance host tolerability and residual tumor clearance when selecting adjuvant regimens for ESCC patients postesophagectomy [35]. Esophagectomy with gastrointestinal reconstruction imposes profound physiological stress on patients. Sustained weight loss—predominantly loss of skeletal muscle and adipose tissue—is universally observed within the first 12 weeks following surgery [36, 37]. Malnutrition and sarcopenia affect a large proportion of survivors throughout the first postoperative year [38, 39], and both conditions correlate with inferior treatment tolerance and poor long‐term survival [40, 41]. Clinically meaningful declines in health‐related quality of life are also well‐documented after esophagectomy [42, 43]. Furthermore, advanced ESCC itself frequently impairs nutritional intake and depletes nutrients reserves [44, 45], an effect compounded by the catabolic toxicity of neoadjuvant therapies [17]. The immediate postoperative window after neoadjuvant therapy and surgical resection therefore represents a period of impaired physical recovery and heightened vulnerability to adjuvant anticancer treatment. In this fragile early recovery phase, many ESCC patients lack sufficient physiological reserve to tolerate adjuvant therapy, which may abrogate potential survival benefits or even worsen clinical outcomes. These detrimental effects are further amplified in underweight patients due to diminished nutritional buffers and compromised treatment tolerability [46]. Only in patients with very advanced pathological disease can the MRD‐suppressing activity of ACT sufficiently outweigh its cumulative toxicity to produce a measurable OS benefit. Single‐agent AIT, by virtue of its superior safety profile, can sustain and amplify the systemic antitumor immunity induced by prior NCIT, delivering durable clinical benefits in eligible postoperative patients.

Several study limitations warrant acknowledgment. Despite our large multicenter real‐world sample, the retrospective noninterventional design introduces inherent selection bias. Unmeasured confounders—including clinician treatment preference and socioeconomic or financial barriers—may have shaped regimen allocation and long‐term patient outcomes. While subgroup analyses identified candidate populations most likely to derive therapeutic benefit, these findings require external prospective validation to confirm generalizability. As this is an exploratory study, the biological and pathological mechanisms underlying the detected clinicopathological parameters impacting benefit from neoadjuvant and adjuvant therapy remain to be clarified. The OTR decision tree models were trained exclusively on clinicopathological variables and lack independent external cohort validation. Incorporating comprehensive pathological and molecular profiles may markedly improve the predictive accuracy of these decision tools, making them worth investigating. Additionally, although the overall cohort size was substantial, certain stratified subgroups contained relatively limited patient numbers, which may reduce statistical power and weaken the reliability of corresponding subgroup conclusions.

## 4 Conclusions

This real‐world cohort study demonstrated comparable long‐term survival across NCT, NCIT, and NCRT for patients with ESCC. Conventional NCT remains a standard perioperative neoadjuvant modality for general ESCC populations. NCIT delivers preferential survival benefits for never‐smokers, nonalcohol abusers, and those presenting with clinical N2–3 nodal disease. NCRT is particularly recommended for obese patients. Postoperatively, AIT sustains therapeutic gains in patients who received prior NCIT, whereas ACT should be reserved for individuals with pathological lymph node metastasis or perineural invasion. We developed OTR decision tree predictive models to facilitate individualized neoadjuvant and adjuvant treatments, which require further validation. These findings provide actionable evidence to support precision perioperative therapy for ESCC patients.

## 5 Methods

### 5.1 Study Design

This retrospective study included ESCC patient who underwent neoadjuvant therapy followed by surgery at four high‐volume esophageal surgery centers in China: The First Affiliated Hospital of Zhengzhou University (January 2019 to June 2023), National Cancer Center/Cancer Hospital (January 2018 to December 2021), Henan Cancer Hospital (January 2020 to December 2022), and Shanxi Cancer Hospital (January 2021 to December 2022). Data were collected prospectively. The study protocol was approved by the Ethics Committee of The First Affiliated Hospital of Zhengzhou University (2025‐KY‐0952‐001). Reporting followed the STROBE Statement [47].

### 5.2 Participants

Consecutive patients diagnosed with ESCC who received neoadjuvant therapy followed by surgery during the study periods were included. Clinical staging was performed according to the eighth edition of the American Joint Committee on Cancer (AJCC) TNM staging system, including T1b‐3N1‐3M0 or T2‐4aN0M0. Neoadjuvant regimens were limited to NCT, NCIT, or NCRT. Surgical approaches included transthoracic esophagectomy. Exclusion criteria included exploration without resection, history of cancer within 5 years, lost to follow‐up, or missing data. For adjuvant therapy analysis, perioperative deaths and early recurrences (<4 weeks postsurgery) were excluded. BMI categories recommended by the World Health Organization for Asian populations were used to classify patients as underwent (<20.0 kg/m^2^), normal (20.0–23.0 kg/m^2^), overweight (23.0–27.5 kg/m^2^), and obese (≥27.5 kg/m^2^) [48].

### 5.3 Treatment Protocols

Across the four centers, NCT for ESCC typically involved two cycles of a platinum‐based doublet, administered every 3 weeks. Platinum agents included cisplatin (75–100 mg/m^2^), nedaplatin (80–100 mg/m^2^), or carboplatin (dosed via the Calvert formula), paired with paclitaxel (135–175 mg/m^2^), docetaxel (75 mg/m^2^), or nab‐paclitaxel (125–150 mg/m^2^). NCIT comprised the same chemotherapy backbone with the addition of a programmed cell death protein 1 (PD‐1) inhibitor—pembrolizumab, nivolumab, camrelizumab, sintilimab, toripalimab, or tislelizumab—across the same two cycles. NCRT involved concurrent chemoradiotherapy, with radiotherapy delivered at a total dose of 40.0–41.4 Gy in 1.8–2.0 Gy fractions 5 days per week, accompanied by weekly chemotherapy over four cycles. Adjuvant therapy, typically commenced 4 weeks postoperatively, was administered according to the neoadjuvant treatment regimen, pathological results, patient physical condition, and individual preference. Adjuvant regimens include ACT, AIT, ART, ACIT, ACRT, and ACIRT. ART, ACRT, and ACIRT were summarized as adjuvant radiotherapies (ARTS) in specific analysis due to small sample sizes.

### 5.4 Endpoints

Primary endpoints were the impact of neoadjuvant therapy on OS and PFS. OS was defined as the time from neoadjuvant therapy initiation to death from any cause. PFS was defined as the time from neoadjuvant therapy initiation to first recurrence/progression or death. Secondary endpoints included the impact of neoadjuvant therapy on perioperative outcomes, pathological results, and 2‐year recurrence patterns. Complications were defined according to the international consensus proposed by the Esophageal Complications Consensus Group [49] and classified by the Clavien‐Dindo system [50]. Pathological staging used the eighth AJCC TNM staging system. CPR was defined as the absence of viable tumor cells in both the primary tumor and all dissected lymph nodes. MPR was defined as <10% residual tumor in the primary site, irrespective of lymph node status. The effect of adjuvant therapy on OS and PFS was further evaluated, with both endpoints calculated from 4 weeks after surgery. Following treatment completion, patients were routinely followed with outpatient visits every 3 months for the first 2 years and every 6 months thereafter.

### 5.5 Statistical Analysis

To compare clinicopathological characteristics, perioperative outcomes, pathological results, and 2‐year recurrence patterns—both across different neoadjuvant therapy groups and between patients with or without adjuvant therapy—we applied the following statistical tests. One‐way ANOVA was used for normally distributed continuous variables. Categorical variables were analyzed using Pearson's chi‐square test or Fisher's exact test. For continuous variables with non‐normal distribution or for ordered categorical variables, the Mann–Whitney *U*‐test (for two‐group comparisons) and the Kruskal–Wallis test (for multiple‐group comparisons) were employed.

Survival analyses were conducted with the Kaplan–Meier method and compared using the log‑rank test. HR and 95%CI were estimated using Cox proportional hazards regression models. PSM was performed using logistic regression at 1:1 or 1:2 ratios, with a caliper width set to 0.2 of the standard deviation of the propensity score. For comparisons among neoadjuvant therapies, matching variables included demographics, comorbidities, pulmonary function, surgical approach, and clinical stage. In the analysis of adjuvant therapy, patients who received adjuvant treatment were matched with those who did not based on demographics, comorbidities, surgical details, neoadjuvant regimen, and pathological outcomes. Subgroup analyses were performed according to clinicopathological parameters to evaluate outcomes in specific populations.

OTR decision trees were constructed using weighted classification and regression trees, with treatment assignment as the outcome and core subgroup variables as predictors [51]. Patient survival benefit scores served as sample weights. The tree was trained with fixed parameters and optimized via cost‐complexity pruning with 10‐fold cross‐validation to avoid overfitting. Internal validation was performed using 1000 bootstrap resamples. Predictive performance was evaluated using the C‑index and 95%CI calculated based on 1000 bootstrap resamples, with values of 0.50–0.59, 0.60–0.69, 0.70–0.79, and ≥0.80 indicating poor, moderate, good, and excellent performance, respectively [52]. The optimal treatment for each subgroup was defined as the most frequent one in each leaf node.

A two‐sided *p* value < 0.05 was considered statistically significant. All statistical analyses were performed using IBM SPSS Statistics for Windows (version 22.0; IBM Corp., Armonk, NY, USA) and R software (version 4.3.1; R Core Team, Vienna, Austria).

## Author Contributions

PW, XL, and YL contributed to study design, data analysis, and data interpretation. XW, YY, ZZ, QG, JL, and WX contributed to data collection and data analysis. All authors contributed to manuscript writing. PW and YL verified the underlying data. All authors had full access to all the data in the study and accept responsibility to submit for publication. All authors read and approved the final manuscript.

## Funding

This work was supported by the National Natural Science Foundation of China (82503220), the Henan Clinical Medical Scientist Program (HNCMS202524), the Postdoctoral Fellowship Program of CPSF (GZB20250537), the China Postdoctoral Science Foundation Science Foundation (2025M772269), and the Key Science and Technology Research Project of Henan Province (252102311029). The funder played no role in study design, data collection, analysis and interpretation of data, or the writing of this manuscript.

## Ethics Statement

This study protocol was approved by the Ethics Committee Board of the First Affiliated Hospital of Zhengzhou University (2025‐KY‐0952‐001). Informed consent was obtained from all patients for the establishment and use of institute databases.

## Conflicts of Interest

The authors declare no conflicts of interest.

## Supporting information




**Supplementary File 1**: mco270909‐sup‐0001‐SuppMat.pdf

## Data Availability

All data generated and analyzed during this study are included in this published article and its Supporting Information. Additional inquiries may be directed to the corresponding authors.
