# Supplementary material for: Advocating for Precision Neoadjuvant and Subsequent Adjuvant Therapies in Esophageal Squamous Cell Carcinoma: Evidence From a Real‐World Multicenter Study
Source: MedComm (2020). 2026 Aug 14;7(8):e70909. doi: 10.1002/mco2.70909 (PMC13473911; doi:10.1002/mco2.70909)
Supplement: Supplementary file 1 [file MCO2-7-e70909-s001.pdf]

Title Page

Title

Advocating for precision neoadjuvant and subsequent adjuvant therapies in esophageal squamous cell carcinoma: evidence from a real-world multicenter study.

Running Title

Precision therapy for esophageal cancer.

Author information and affiliation

Peiyu Wang, MD, PhD [1]*; Xiufeng Wei, MD [2]*; Yongkui Yu, MD, PhD [3]*; Zhiqian Zhao, MD [1]*; Qiuchen Ge, MD [4]*; Jianhong Lian, MD [4]; Wenqun Xing, MD [3]; Xiangnan Li, MD, PhD [1]; Yin Li, MD, PhD [2]

[1] Department of Thoracic Surgery, The First Affiliated Hospital of Zhengzhou University, Zhengzhou, Henan 450052, China.

[2] Department of Thoracic Surgical Oncology, National Cancer Center/Cancer Hospital, Chinese Academy of Medical Sciences and Peking Union Medical College, Beijing, 100021, China

[3] Department of Thoracic Surgery, The Affiliated Cancer Hospital of Zhengzhou University, Henan Cancer Hospital, Zhengzhou, Henan 450000, China

[4] Department of Thoracic Surgery, Shanxi Province Cancer Hospital/Shanxi Hospital Affiliated to Cancer Hospital, Chinese Academy of Medical Sciences/Cancer Hospital Affiliated to Shanxi Medical University, Taiyuan, Shanxi 030000, China.

* These authors contributed equally.

Corresponding authors:

Peiyu Wang (Lead contact)

Department of Thoracic Surgery, The First Affiliated Hospital of Zhengzhou University, Zhengzhou, Henan 450052, China.

Email: wpy_surgery@zzu.edu.cn; ORCID: 0000-0002-7021-3939.

Xiangnan Li

Department of Thoracic Surgery, The First Affiliated Hospital of Zhengzhou University, Zhengzhou, Henan 450052, China.

Email: fcclixn@zzu.edu.cn; ORCID: 0000-0002-2236-8418.

Yin Li

Department of Thoracic Surgical Oncology, National Cancer Center/Cancer Hospital, Chinese Academy of Medical Sciences and Peking Union Medical College, Beijing, 100021, China.

Email: liyin@cicams.ac.cn; ORCID: 0000-0002-7840-5548.

List of supplementary materials

Table S1: pg.3

Table S2: pg.4

Table S3: pg.5

Table S4: pg.6

Table S5: pg.7-8

Figure S1: pg.9

Figure S2: pg.10

Figure S3: pg.11

Figure S4: pg.12

Figure S5: pg.13

Figure S6: pg.14

Figure S7: pg.15

Figure S8: pg.16

Table S1. Baseline characteristics by neoadjuvant therapy after propensity score matching.

Characteristics	NCT vs. NCIT (ratio 1:1)			NCT vs. NCRT (ratio 1:1)			NCIT vs. NCRT (ratio 2:1)		
	NCT ($N = 576$)	NCIT ($N = 576$)	P value	NCT ($N = 393$)	NCRT ($N = 393$)	P value	NCIT ($N = 592$)	NCRT ($N = 379$)	P value
Demographics									
Age, year	64.1 ± 8.1	63.9 ± 7.8	0.75	62.7 ± 8.1	62.9 ± 8.0	0.79	63.5 ± 7.9	63.0 ± 7.6	0.32
Gender									
Male	430 (74.7)	423 (73.4)	0.64	315 (80.2)	317 (80.7)	0.86	459 (77.5)	309 (81.5)	0.14
Female	146 (25.3)	153 (26.6)		78 (19.8)	76 (19.3)		133 (22.5)	70 (18.5)	
Smoking history									
Yes	315 (54.7)	308 (53.5)	0.68	228 (58.0)	228 (58.0)	1.00	314 (53.0)	217 (57.3)	0.20
No	261 (45.3)	268 (46.5)		165 (42.0)	165 (42.0)		278 (47.0)	162 (42.7)	
Alcohol misuse									
Yes	244 (42.4)	246 (42.7)	0.91	196 (49.9)	203 (51.7)	0.62	288 (48.6)	199 (52.5)	0.24
No	332 (57.6)	330 (57.3)		197 (50.1)	190 (48.3)		304 (51.4)	180 (47.5)	
Body mass index, kg/m^2	23.7 ± 3.3	23.4 ± 3.3	0.22	23.4 ± 3.3	23.4 ± 3.2	0.96	23.3 ± 3.3	23.3 ± 3.1	0.94
Comorbidities									
Cardiovascular disease									
Yes	78 (13.5)	79 (13.7)	0.93	50 (12.7)	46 (11.7)	0.66	79 (13.3)	46 (12.1)	0.58
No	498 (86.5)	497 (86.3)		343 (87.3)	347 (88.3)		513 (86.7)	333 (87.9)	
Pulmonary disease									
Yes	18 (3.1)	16 (2.8)	0.73	13 (3.3)	15 (3.8)	0.70	15 (2.5)	14 (3.7)	0.30
No	558 (96.9)	560 (97.2)		380 (96.7)	378 (96.2)		577 (97.5)	365 (96.3)	
Diabetes									
Yes	63 (10.9)	59 (10.2)	0.70	44 (11.2)	45 (11.5)	0.91	58 (9.8)	43 (11.3)	0.44
No	513 (89.1)	517 (89.8)		349 (88.8)	348 (88.5)		534 (90.2)	336 (88.7)	
Charlson comorbidity index									
< 3	419 (72.7)	428 (74.3)	0.55	284 (72.3)	289 (73.5)	0.69	146 (24.7)	102 (26.9)	0.43
≥ 3	157 (27.3)	148 (25.7)		109 (27.7)	104 (26.5)		446 (75.3)	277 (73.1)	
Pulmonary function									
FEV1.0, L	2.60 (2.18-3.03)	2.62 (2.17-3.07)	0.73	2.69 (2.28-3.11)	2.60 (2.25-3.17)	0.45	2.68 (2.19-3.09)	2.62 (2.26-3.20)	0.91
DLCO-SB, mmol/min/kPa	6.94 (5.89-7.97)	6.93 (5.90-8.08)	0.86	7.06 (6.03-8.18)	7.13 (5.96-8.21)	0.65	6.93 (5.96-7.95)	7.13 (5.99-8.20)	0.15
Surgical parameters									
Surgery approach									
McKeown	553 (96.0)	553 (96.0)	0.80	384 (97.7)	384 (97.7)	0.61	579 (97.8)	371 (97.9)	0.87
Ivor Lewis	18 (3.1)	16 (2.8)		7 (1.8)	5 (1.3)		8 (1.4)	4 (1.1)	
Sweet	5 (0.9)	7 (1.2)		2 (0.5)	4 (1.0)		5 (0.8)	4 (1.1)	
Surgery technique									
VATS			0.26			0.65			0.61
Robot assisted surgery	24 (4.2)	14 (2.4)		14 (3.6)	17 (4.3)		23 (3.9)	17 (4.5)	
Thoracotomy	13 (2.3)	13 (2.3)		25 (6.4)	20 (5.1)		12 (2.0)	11 (2.9)	
Tumor characteristics									
Tumor location									
Proximal third	72 (12.6)	81 (14.1)	0.90	46 (11.9)	77 (20.1)	0.26	85 (14.4)	74 (20.1)	0.50
Middle third	287 (50.4)	271 (47.3)		197 (50.8)	159 (41.5)		287 (48.7)	154 (41.7)	
Distal third	211 (37.0)	221 (38.6)		145 (37.4)	147 (38.4)		217 (36.8)	141 (38.2)	
Clinical T stage									
cT1	66 (11.5)	80 (13.9)	0.61	37 (9.4)	50 (12.7)	0.13	77 (13.0)	50 (13.2)	0.17
cT2	109 (18.9)	78 (13.5)		67 (17.0)	41 (10.4)		75 (12.7)	40 (10.6)	
cT3	385 (66.8)	405 (70.3)		272 (69.2)	268 (68.2)		429 (72.5)	269 (71.0)	
cT4	16 (2.8)	13 (2.3)		17 (4.3)	34 (8.7)		11 (1.9)	20 (5.3)	
Clinical N stage									
cN0	242 (42.0)	250 (43.4)	0.92	152 (38.7)	153 (38.9)	0.37	267 (45.1)	145 (38.3)	0.71
cN1	228 (39.6)	204 (35.4)		172 (43.8)	189 (48.1)		203 (34.3)	181 (47.8)	
cN2	86 (14.9)	104 (18.1)		57 (14.5)	51 (13.0)		102 (17.2)	53 (14.0)	
cN3	20 (3.5)	18 (3.1)		12 (3.1)	0		20 (3.4)	0	
Clinical TNM stage									
I	60 (10.4)	62 (10.8)	0.82	31 (7.9)	41 (10.4)	0.64	56 (9.5)	41 (10.8)	0.23
II	263 (45.7)	252 (43.8)		165 (42.0)	145 (36.9)		264 (44.6)	142 (37.5)	
III	219 (38.0)	233 (40.5)		170 (43.3)	173 (44.0)		241 (40.7)	176 (46.4)	
IV	34 (5.9)	29 (5.0)		27 (6.9)	34 (8.7)		31 (5.2)	20 (5.3)	

Data are presented as mean ± standard deviation, number (percentage), or median (interquartile range).

Group differences were assessed using ANOVA, Pearson's chi-squared tests, Fisher's exact tests, or Mann–Whitney U tests.

Abbreviations: DLCO-SB, single-breath diffusing capacity of the lung for carbon monoxide; FEV1, forced expiratory volume in 1 second; NCIT, neoadjuvant chemoimmunotherapy; NCRT, neoadjuvant chemoradiotherapy; NCT, neoadjuvant chemotherapy; VATS, video-assisted thoracic surgery.

Table S2. Short-term outcomes by neoadjuvant therapy.

Characteristics	Neoadjuvant therapies			*P-total*	*P1*	*P2*	*P3*
	NCT (*N* = 652)	NCIT (*N* = 765)	NCRT (*N* = 401)				
Intraoperative outcomes							
Operation time, min	280 (240-330)	285 (235-340)	280 (235-328)	0.24	0.71	0.19	0.10
Estimated blood loss, ml	100 (50-200)	100 (50-200)	100 (50-200)	0.65	0.33	0.26	0.37
Lymph node dissection, No.	27 (19-35)	30 (22-40)	24 (17-33)	<0.001	<0.001	0.001	<0.001
Radicality of surgery							
R0	650 (99.7)	759 (99.2)	393 (98.0)	0.016	0.40	0.016	0.13
R1	2 (0.3)	6 (0.8)	8 (2.0)				
Postoperative complications							
Specific complications							
Pneumonia	111 (17.0)	110 (14.4)	56 (14.0)	0.28	0.17	0.19	0.85
Pleural effusion	81 (12.4)	126 (16.5)	60 (15.0)	0.099	0.032	0.24	0.50
Pneumothorax	7 (1.1)	14 (1.8)	6 (1.5)	0.50	0.24	0.75	0.68
Respiratory failure	10 (1.5)	14 (1.8)	1 (0.2)	0.081	0.67	0.093	0.023
Myocardial arrhythmia	5 (0.8)	14 (1.8)	6 (1.5)	0.22	0.083	0.41	0.68
Congestive heart failure	3 (0.5)	4 (0.5)	3 (0.7)	0.82	0.87	0.86	0.64
Anastomotic leak	34 (5.2)	55 (7.2)	30 (7.5)	0.23	0.13	0.14	0.86
Wound infection	12 (1.8)	10 (1.3)	5 (1.2)	0.64	0.42	0.46	0.93
Chylothorax	2 (0.3)	4 (0.5)	0	0.33	0.53	0.53	0.36
Overall complications	166 (25.5)	215 (28.1)	94 (23.4)	0.20	0.26	0.46	0.087
Clavien–Dindo complication grades							
I	12 (1.8)	17 (2.2)	7 (1.7)	0.16	0.20	0.49	0.072
II	78 (12.0)	80 (10.5)	37 (9.2)				
IIIA	54 (8.3)	94 (12.3)	46 (11.5)				
IIIB	6 (0.9)	7 (0.9)	0				
IV	12 (1.8)	13 (1.7)	2 (0.5)				
V	5 (0.8)	4 (0.5)	2 (0.5)				
Postoperative hospital stay, days	9 (8-12)	10 (8-13)	10 (8-12)	0.078	0.058	0.75	0.053
Pathological outcomes							
Complete pathologic response							
Yes	63 (9.7)	190 (24.8)	139 (34.7)	<0.001	<0.001	<0.001	<0.001
No	589 (90.3)	575 (75.2)	262 (65.3)				
Major pathological response							
Yes	142 (21.8)	338 (44.2)	264 (65.8)	<0.001	<0.001	<0.001	<0.001
No	510 (78.2)	427 (55.8)	137 (34.2)				
ypT stage							
ypT0	84 (12.9)	219 (28.6)	175 (43.6)	<0.001	<0.001	<0.001	<0.001
ypT1	116 (17.8)	150 (19.6)	59 (14.7)				
ypT2	177 (27.1)	194 (25.4)	74 (18.5)				
ypT3	266 (40.8)	195 (25.5)	89 (22.2)				
ypT4	9 (1.4)	7 (0.9)	4 (1.0)				
ypN stage							
ypN0	351 (53.8)	458 (59.9)	284 (70.8)	<0.001	0.026	<0.001	0.001
ypN1	182 (27.9)	190 (24.8)	81 (20.2)				
ypN2	93 (14.3)	84 (11.0)	30 (7.5)				
ypN3	26 (4.0)	33 (4.3)	6 (1.5)				
ypTNM stage							
ypI	245 (37.6)	383 (50.1)	236 (58.9)	<0.001	<0.001	<0.001	<0.001
ypII	103 (15.8)	71 (9.3)	46 (11.5)				
ypIIIA	89 (13.7)	122 (15.9)	52 (13.0)				
ypIIIB	183 (28.1)	153 (20.0)	59 (14.7)				
ypIVA	32 (4.9)	36 (4.7)	8 (2.0)				
Lymphovascular invasion							
Yes	186 (28.5)	146 (19.1)	50 (12.5)	<0.001	<0.001	<0.001	0.004
No	466 (71.5)	619 (80.9)	351 (87.5)				
Perineural invasion							
Yes	140 (21.5)	101 (13.2)	45 (11.2)	<0.001	<0.001	<0.001	0.33
No	512 (78.5)	664 (86.8)	356 (88.8)				
Adjuvant therapy							
Yes	368 (57.1)	403 (53.0)	139 (35.0)	<0.001	0.12	<0.001	<0.001
No	276 (42.9)	357 (47.0)	258 (65.0)				

Data are presented as number (percentage) or median (interquartile range).

Group differences were assessed using Pearson's chi-squared test, Fisher's exact test, Mann–Whitney U test, or Kruskal–Wallis test. P values are denoted as follows: P-total (comparison among NCT, NCIT, and NCRT groups), P1 (NCIT vs. NCT), P2 (NCRT vs. NCT), and P3 (NCIT vs. NCRT).

Abbreviations: NCIT, neoadjuvant chemoimmunotherapy; NCRT, neoadjuvant chemoradiotherapy; NCT, neoadjuvant chemotherapy.

Table S3. Two-year recurrence by neoadjuvant therapy.

Characteristics	Neoadjuvant therapies			*P-total*	*P1*	*P2*	*P3*
	NCT (*N* = 652)	NCIT (*N* = 765)	NCRT (*N* = 401)				
Overall recurrence							
Yes	118 (18.1)	152 (19.9)	78 (19.5)	0.69	0.40	0.58	0.87
No	534 (81.9)	613 (80.1)	323 (80.5)				
Locoregional recurrence							
Yes	62 (9.5)	78 (10.2)	32 (8.0)	0.47	0.67	0.40	0.22
No	590 (90.5)	687 (89.8)	369 (92.0)				
Distant metastasis							
Yes	95 (14.6)	122 (15.9)	67 (16.7)	0.62	0.47	0.35	0.74
No	557 (85.4)	643 (84.1)	334 (83.3)				
Combined recurrence							
Yes	39 (6.0)	48 (6.3)	21 (5.2)	0.78	0.82	0.61	0.48
No	613 (94.0)	717 (93.7)	380 (94.8)				
Specific classifications of recurrence							
Regional lymph nodes							
Yes	42 (6.4)	57 (7.5)	19 (4.7)	0.20	0.46	0.25	0.075
No	610 (93.6)	708 (92.5)	382 (95.3)				
Anastomosis recurrence							
Yes	27 (4.1)	29 (3.8)	16 (4.0)	0.94	0.74	0.90	0.87
No	625 (95.9)	736 (96.2)	385 (96.0)				
Supraclavicular lymph node metastasis							
Yes	25 (3.8)	27 (3.5)	11 (2.7)	0.64	0.76	0.34	0.47
No	627 (96.2)	738 (96.5)	390 (97.3)				
Distant organs metastasis							
Yes	77 (11.8)	103 (13.5)	65 (16.2)	0.13	0.35	0.042	0.21
No	575 (88.2)	662 (86.5)	336 (83.8)				

Data are presented as number (percentage).

Group differences were assessed using Pearson's chi-squared tests. P values are denoted as follows: P-total (comparison among NCT, NCIT, and NCRT groups), P1 (NCIT vs. NCT), P2 (NCRT vs. NCT), and P3 (NCIT vs. NCRT).

Abbreviations: NCIT, neoadjuvant chemoimmunotherapy; NCRT, neoadjuvant chemoradiotherapy; NCT, neoadjuvant chemotherapy.

Table S4. Two-year recurrence by neoadjuvant therapy after propensity score matching.

Characteristics	NCT vs. NCIT (ratio 1:1)			NCT vs. NCRT (ratio 1:1)			NCIT vs. NCRT (ratio 2:1)		
	NCT (N = 576)	NCIT (N = 576)	P value	NCT (N = 393)	NCRT (N = 393)	P value	NCIT (N = 592)	NCRT (N = 379)	P value
Overall recurrence									
Yes	103 (17.9)	114 (19.8)	0.41	82 (20.9)	77 (19.6)	0.66	114 (19.3)	72 (19.0)	0.92
No	473 (82.1)	462 (80.2)		311 (79.1)	316 (80.4)		478 (80.7)	307 (81.0)	
Locoregional recurrence									
Yes	54 (9.4)	5 (0.9)	0.69	43 (10.9)	31 (7.9)	0.14	58 (9.8)	26 (6.9)	0.11
No	522 (90.6)	518 (89.9)		350 (89.1)	362 (92.1)		534 (90.2)	353 (93.1)	
Distant metastasis									
Yes	84 (14.6)	93 (16.1)	0.46	65 (16.5)	67 (17.0)	0.85	94 (15.9)	62 (16.4)	0.84
No	492 (85.4)	483 (83.9)		328 (83.5)	326 (83.0)		498 (84.1)	317 (83.6)	
Combined recurrence									
Yes	35 (6.1)	37 (6.4)	0.81	26 (6.6)	21 (5.3)	0.45	38 (6.4)	16 (4.2)	0.15
No	541 (93.9)	539 (93.6)		367 (93.4)	372 (94.7)		554 (93.6)	363 (95.8)	
Specific classifications of recurrence									
Regional lymph nodes									
Yes	35 (6.1)	41 (7.1)	0.48	28 (7.1)	18 (4.6)	0.13	43 (7.3)	15 (4.0)	0.034
No	541 (93.9)	535 (92.9)		365 (92.9)	375 (95.4)		549 (92.7)	364 (96.0)	
Anastomosis recurrence									
Yes	25 (4.3)	22 (3.8)	0.66	20 (5.1)	15 (3.8)	0.39	23 (3.9)	13 (3.4)	0.71
No	551 (95.7)	554 (96.2)		373 (94.9)	378 (96.2)		569 (96.1)	366 (96.6)	
Supraclavicular lymph node metastasis									
Yes	23 (4.0)	18 (3.1)	0.43	20 (5.1)	11 (2.8)	0.099	20 (3.4)	8 (2.1)	0.25
No	553 (96.0)	558 (96.9)		373 (94.9)	382 (97.2)		572 (96.6)	371 (97.9)	
Distant organs metastasis									
Yes	67 (11.6)	79 (13.7)	0.29	50 (12.7)	65 (16.5)	0.13	80 (13.5)	61 (16.1)	0.27
No	509 (88.4)	497 (86.3)		343 (87.3)	328 (83.5)		512 (86.5)	318 (83.9)	

Data are presented as number (percentage).

Group differences were assessed using Pearson's chi-squared test or Fisher's exact test.

Abbreviations: NCIT, neoadjuvant chemoimmunotherapy; NCRT, neoadjuvant chemoradiotherapy; NCT, neoadjuvant chemotherapy.

Table S5. Baseline characteristics by adjuvant therapy.

Characteristics	Administration of adjuvant therapy after surgery					
	Before propensity score matching			After propensity score matching		
	No (N = 891)	Yes (N = 910)	P value	No (N = 695)	Yes (N = 695)	P value
Demographics						
Age, year	64.5 ± 7.8	62.9 ± 8.0	<0.001	63.8 ± 7.8	63.6 ± 7.9	0.57
Gender						
Male	678 (76.1)	701 (77.0)	0.64	528 (76.0)	524 (75.4)	0.80
Female	213 (23.9)	209 (23.0)		167 (24.0)	171 (24.6)	
Smoking history						
Yes	491 (55.1)	494 (54.3)	0.73	374 (53.8)	380 (54.7)	0.75
No	400 (44.9)	416 (45.7)		321 (46.2)	315 (45.3)	
Alcohol misuse						
Yes	398 (44.7)	416 (45.7)	0.66	304 (43.7)	313 (45.0)	0.63
No	493 (55.3)	494 (54.3)		391 (56.3)	382 (55.0)	
Body mass index, kg/m^2	23.2 ± 3.2	23.6 ± 3.3	0.020	23.4 ± 3.2	23.4 ± 3.4	0.97
Comorbidities						
Cardiovascular disease						
Yes	111 (12.5)	112 (12.3)	0.92	84 (12.1)	83 (11.9)	0.93
No	780 (87.5)	798 (87.7)		611 (87.9)	612 (88.1)	
Pulmonary disease						
Yes	33 (3.7)	24 (2.6)	0.20	22 (3.2)	20 (2.9)	0.75
No	858 (96.3)	886 (97.4)		673 (96.8)	675 (97.1)	
Diabetes						
Yes	105 (11.8)	80 (8.8)	0.036	74 (10.6)	63 (9.1)	0.32
No	786 (88.2)	830 (91.2)		621 (89.4)	632 (90.9)	
Charlson comorbidity index						
< 3	653 (73.3)	687 (75.5)	0.28	522 (75.1)	519 (74.7)	0.85
≥ 3	238 (26.7)	223 (24.5)		173 (24.9)	176 (25.3)	
Surgical parameters						
Tumor location						
Proximal third	140 (15.7)	126 (13.8)	0.70	106 (15.3)	98 (14.1)	0.58
Middle third	402 (45.1)	452 (49.7)		316 (45.5)	344 (49.5)	
Distal third	338 (37.9)	323 (35.5)		264 (38.0)	246 (35.4)	
Surgery approach						
McKeown	847 (95.1)	868 (95.4)	0.75	663 (95.4)	661 (95.1)	0.80
Ivor Lewis/ Sweet	44 (4.9)	42 (4.6)		32 (4.6)	34 (4.9)	
Surgery technique						
VATS/RAT	848 (95.2)	862 (94.7)	0.66	660 (95.0)	660 (95.0)	1.00
Thoracotomy	43 (4.8)	48 (5.3)		35 (5.0)	35 (5.0)	
Radicality of surgery						
R0	884 (99.2)	903 (99.2)	0.97	690 (99.3)	690 (99.3)	1.00
R1	7 (0.8)	7 (0.8)		5 (0.7)	5 (0.7)	
Overall complications						
Yes	243 (27.3)	221 (24.3)	0.15	171 (24.6)	182 (26.2)	0.50
No	648 (72.7)	689 (75.7)		524 (75.4)	513 (73.8)	
Clavien–Dindo complication grades						
I	19 (2.1)	17 (1.9)	0.084	13 (1.9)	13 (1.9)	0.62
II	89 (10.0)	105 (11.5)		68 (9.8)	88 (12.7)	
IIIA	113 (12.7)	81 (8.9)		77 (11.1)	67 (9.6)	
IIIB	8 (0.9)	5 (0.5)		4 (0.6)	3 (0.4)	
IV	14 (1.6)	13 (1.4)		9 (1.3)	11 (1.6)	
Neoadjuvant therapy						
NCT	276 (31.0)	368 (40.4)	<0.001	254 (36.5)	249 (35.8)	0.39
NCIT	357 (40.1)	403 (44.3)		295 (42.4)	317 (45.6)	
NCRT	258 (29.0)	139 (15.3)		146 (21.0)	129 (18.6)	
Pathological outcomes						
Complete pathological response						
Yes	257 (58.8)	133 (14.6)	<0.001	140 (21.4)	131 (18.8)	0.54
No	634 (71.2)	777 (85.4)		555 (78.6)	564 (81.2)	
Major pathological response						
Yes	430 (48.3)	310 (34.1)	<0.001	278 (40.0)	277 (39.9)	0.96
No	461 (51.7)	600 (65.9)		417 (60.0)	418 (60.1)	

ypT category						
ypT0	287 (32.2)	188 (20.7)	<0.001	177 (25.5)	172 (24.7)	0.54
ypT1	174 (19.5)	150 (16.5)		138 (19.9)	115 (16.5)	
ypT2	203 (22.8)	237 (26.0)		163 (23.5)	192 (27.6)	
ypT3	218 (24.5)	324 (35.6)		208 (29.9)	210 (30.2)	
ypT4	9 (1.0)	11 (1.2)		9 (1.3)	6 (0.9)	
ypN category						
ypN0	636 (71.4)	448 (49.2)	<0.001	446 (64.2)	417 (60.0)	0.14
ypN1	164 (18.4)	285 (31.3)		160 (23.0)	181 (26.0)	
ypN2	70 (7.9)	133 (14.6)		69 (9.9)	81 (11.7)	
ypN3	21 (2.4)	44 (4.8)		20 (2.9)	16 (2.3)	
ypTNM stage						
ypI	523 (58.7)	335 (36.8)	<0.001	342 (49.2)	321 (46.2)	0.28
ypII	107 (12.0)	110 (12.1)		98 (14.1)	93 (13.4)	
ypIIIA	99 (11.1)	161 (17.7)		95 (13.7)	113 (16.3)	
ypIIIB	137 (15.4)	253 (27.8)		136 (19.6)	150 (21.6)	
ypIVA	25 (2.8)	51 (5.6)		24 (3.5)	18 (2.6)	
Lymphovascular invasion						
Yes	143 (16.0)	236 (25.9)	<0.001	134 (19.3)	144 (20.7)	0.50
No	748 (84.0)	674 (74.1)		561 (80.7)	551 (79.3)	
Perineural invasion						
Yes	105 (11.8)	178 (19.6)	<0.001	99 (14.2)	113 (16.3)	0.30
No	786 (88.2)	732 (80.4)		596 (85.8)	582 (83.7)	

Data are presented as mean ± standard deviation, number (percentage), or median (interquartile range).

Group differences were assessed using ANOVA, Pearson's chi-squared test, Fisher's exact test, or Mann–Whitney U test.

Abbreviations: NCIT, neoadjuvant chemoimmunotherapy; NCRT, neoadjuvant chemoradiotherapy; NCT, neoadjuvant chemotherapy; RAT, robot assisted surgery; VATS, video assisted thoracic surgery.

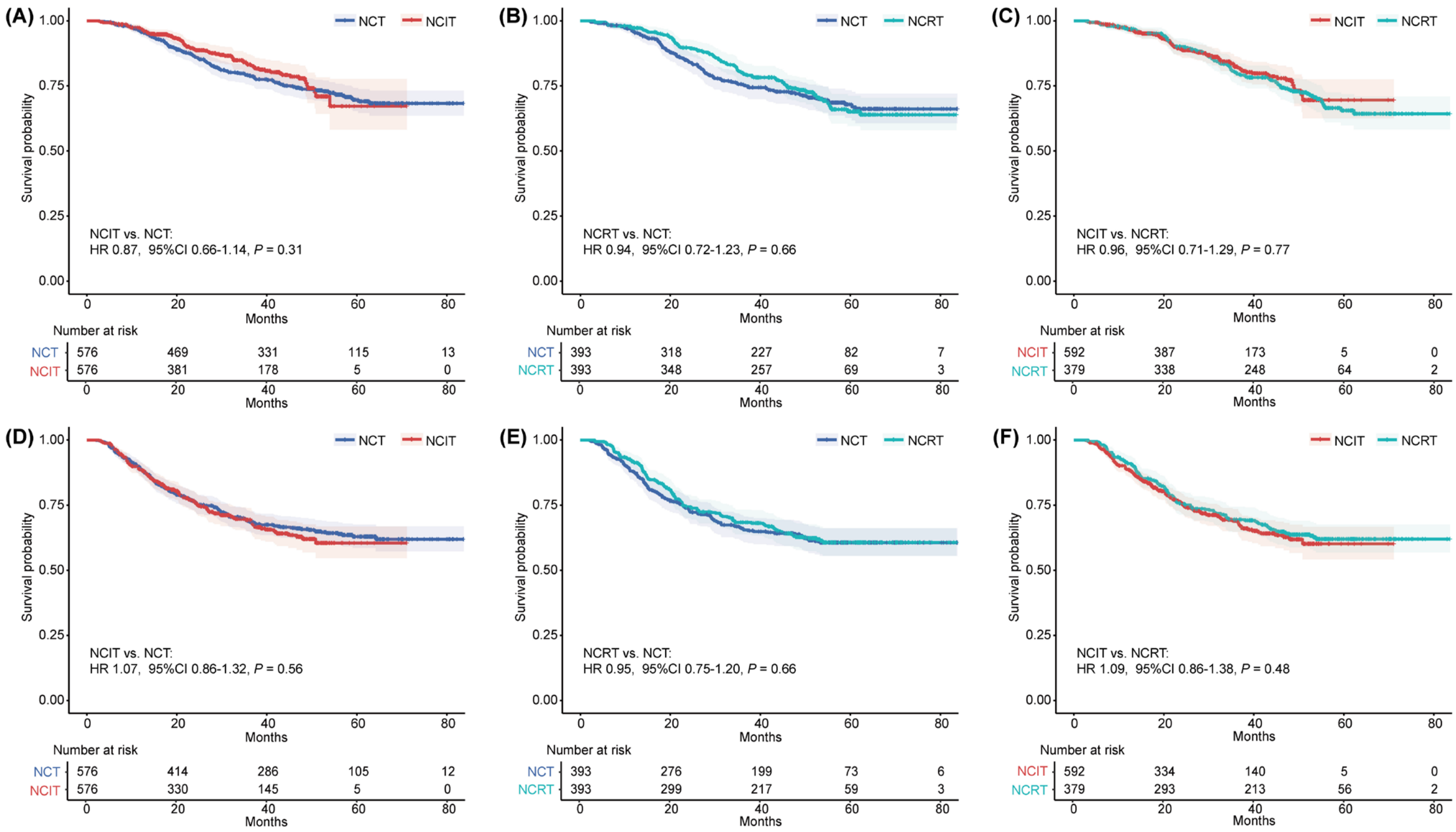

Figure S1: Overall survival (OS) and progression-free survival (PFS) by neoadjuvant therapy after propensity score matching (PSM).

A-C: OS comparisons after PSM: NCT vs. NCIT (A), NCT vs. NCRT (B), and NCIT vs. NCRT (C).

D-F: PFS comparisons after PSM: NCT vs. NCIT (D), NCT vs. NCRT (E), and NCIT vs. NCRT (F).

Abbreviations: CI, confidence interval; HR, hazard ratio; NCIT, neoadjuvant chemoimmunotherapy; NCRT, neoadjuvant chemoradiotherapy; NCT, neoadjuvant chemotherapy; PSM, propensity score matching.

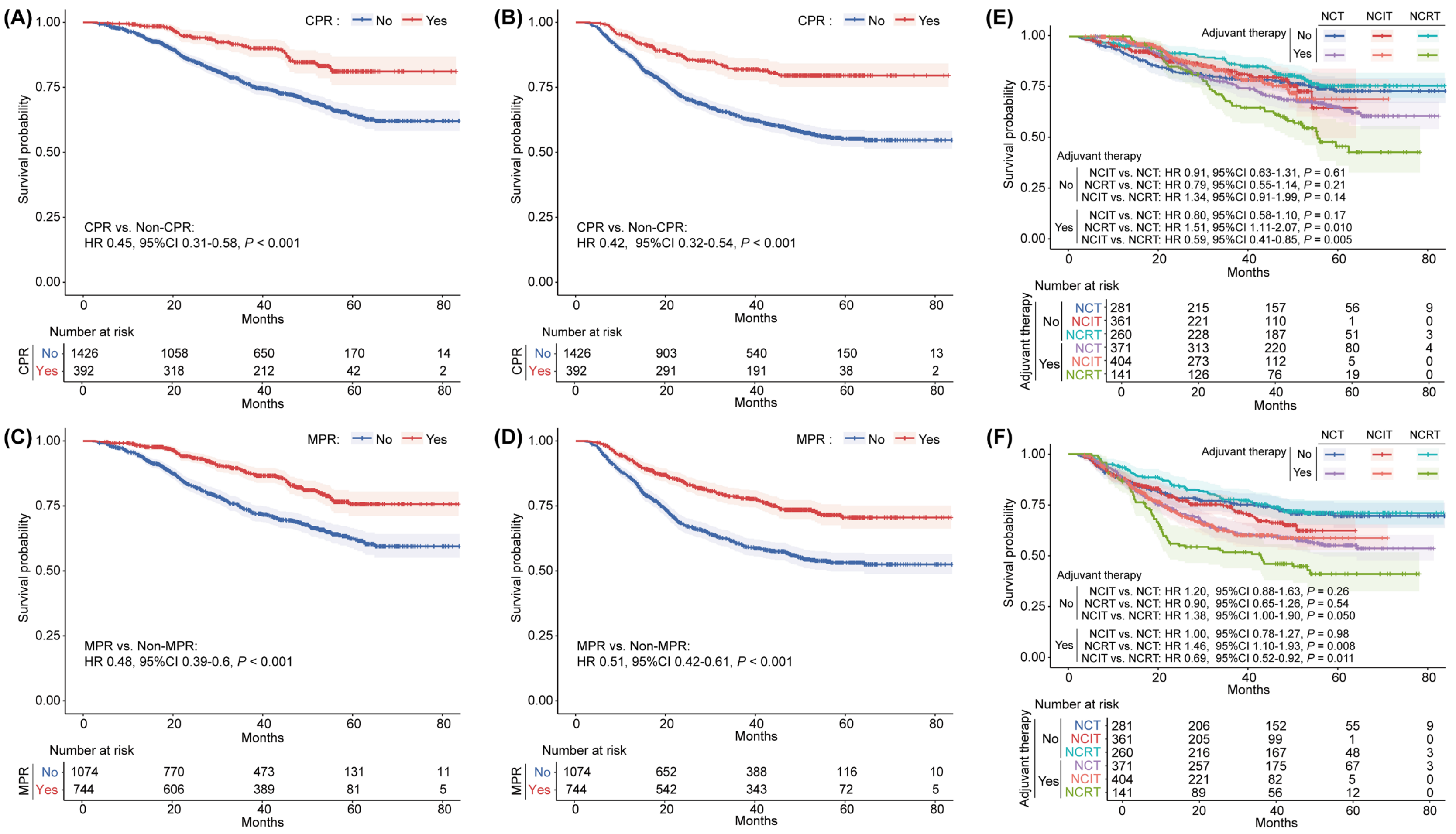

Figure S2. Analysis of overall survival (OS) and progression-free survival (PFS) by pathological outcomes.

A-B: OS (A) and PFS (B) by CPR status after neoadjuvant therapy.

C-D: OS (C) and PFS (D) by MPR status after neoadjuvant therapy.

E-F: OS (E) and PFS (F) for patients receiving NCT, NCIT, or NCRT, stratified by receipt of adjuvant therapy.

Abbreviations: CI, confidence interval; CPR, Complete pathological response; HR, hazard ratio; MPR, major pathological response; NCIT, neoadjuvant chemoimmunotherapy; NCRT, neoadjuvant chemoradiotherapy; NCT, neoadjuvant chemotherapy.

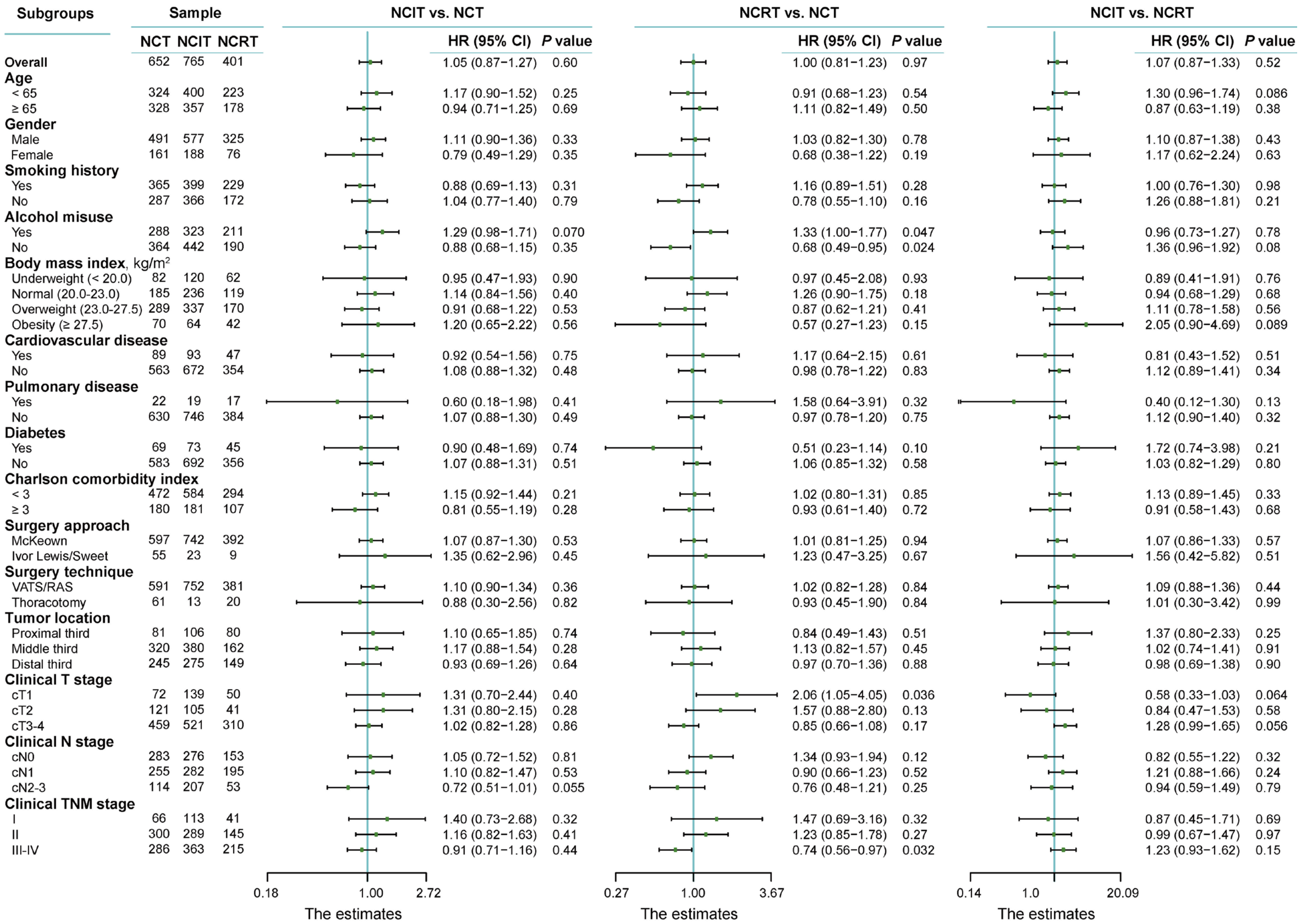

Figure S3. Subgroup analysis of progression-free survival by neoadjuvant therapy. Forest plot showing hazard ratios for progression-free survival among patients treated with different neoadjuvant therapies followed by surgery.

Abbreviations: CI, confidence interval; HR, hazard ratio; NCIT, neoadjuvant chemoimmunotherapy; NCRT, neoadjuvant chemoradiotherapy; NCT, neoadjuvant chemotherapy; RAT, robot-assisted surgery; VATS, video-assisted thoracic surgery.

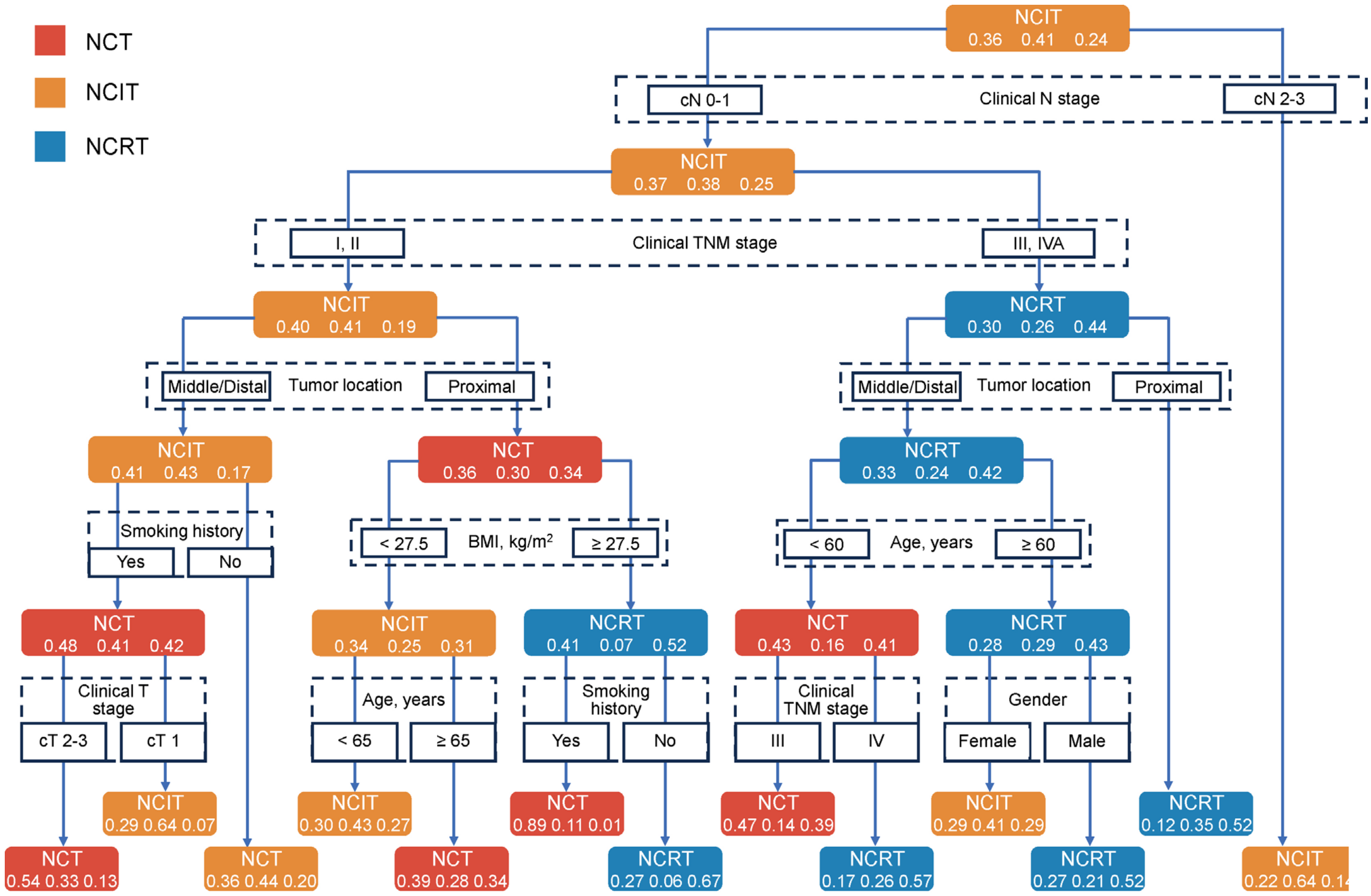

Figure S4. Optimal treatment rule (OTR) decision tree for selecting neoadjuvant therapies based on progression-free survival, using core subgroup variables as predictors. The three values in each box represent, from left to right, the probability of benefit from neoadjuvant chemotherapy (NCT), neoadjuvant chemoimmunotherapy (NCIT), and neoadjuvant chemoradiotherapy (NCRT).

Abbreviations: BMI, body mass index; NCIT, neoadjuvant chemoimmunotherapy; NCRT, neoadjuvant chemoradiotherapy; NCT, neoadjuvant chemotherapy.

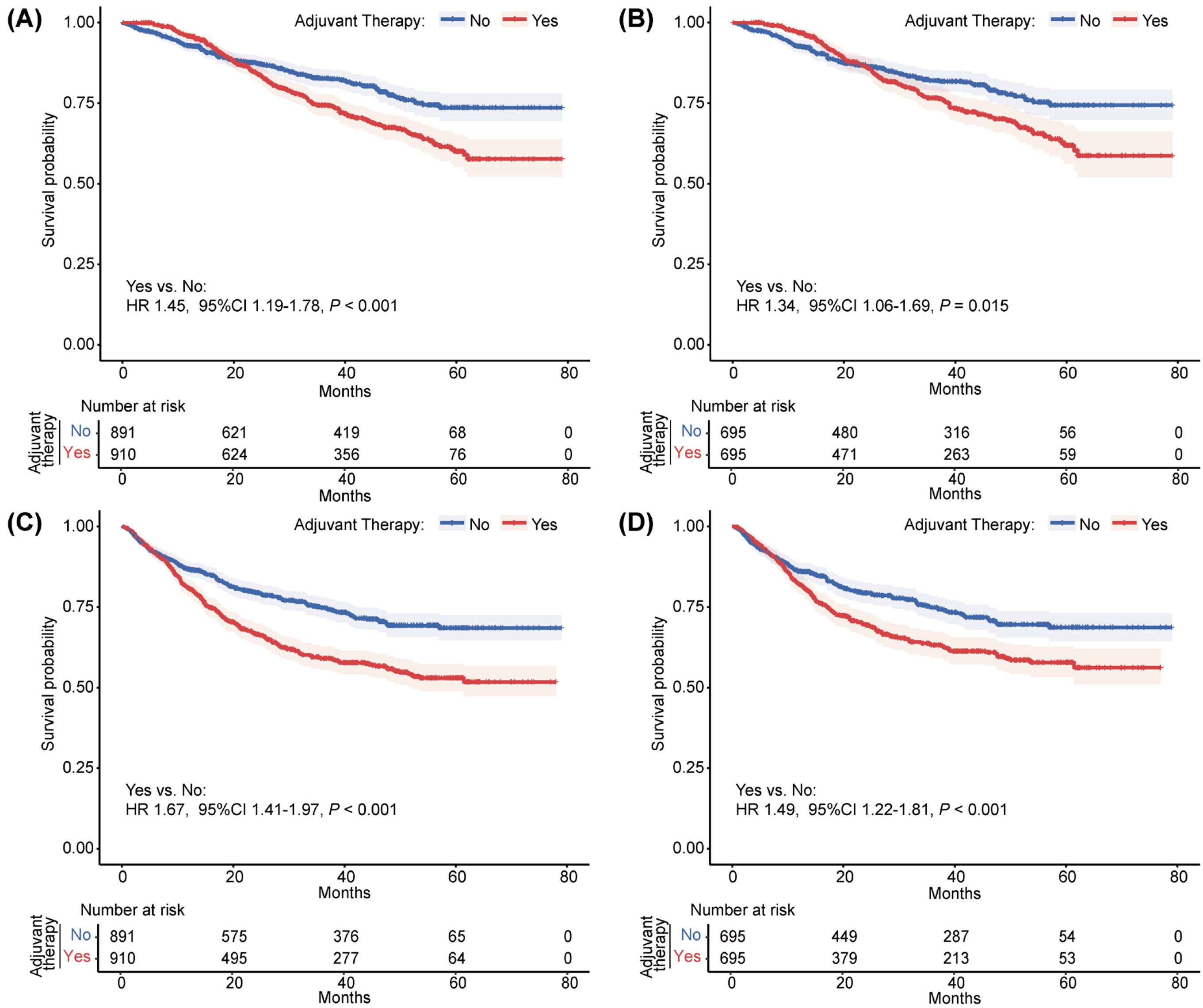

Figure S5. Overall survival (OS) and progression-free survival (PFS) by adjuvant therapy.

A-B: OS comparing patients who did or did not receive adjuvant therapy, before PSM (A) and after PSM (B).

C-D: PFS comparing patients who did or did not receive adjuvant therapy, before PSM (C) and after PSM (D).

Abbreviations: CI, confidence interval; HR, hazard ratio; PSM, propensity score matching.

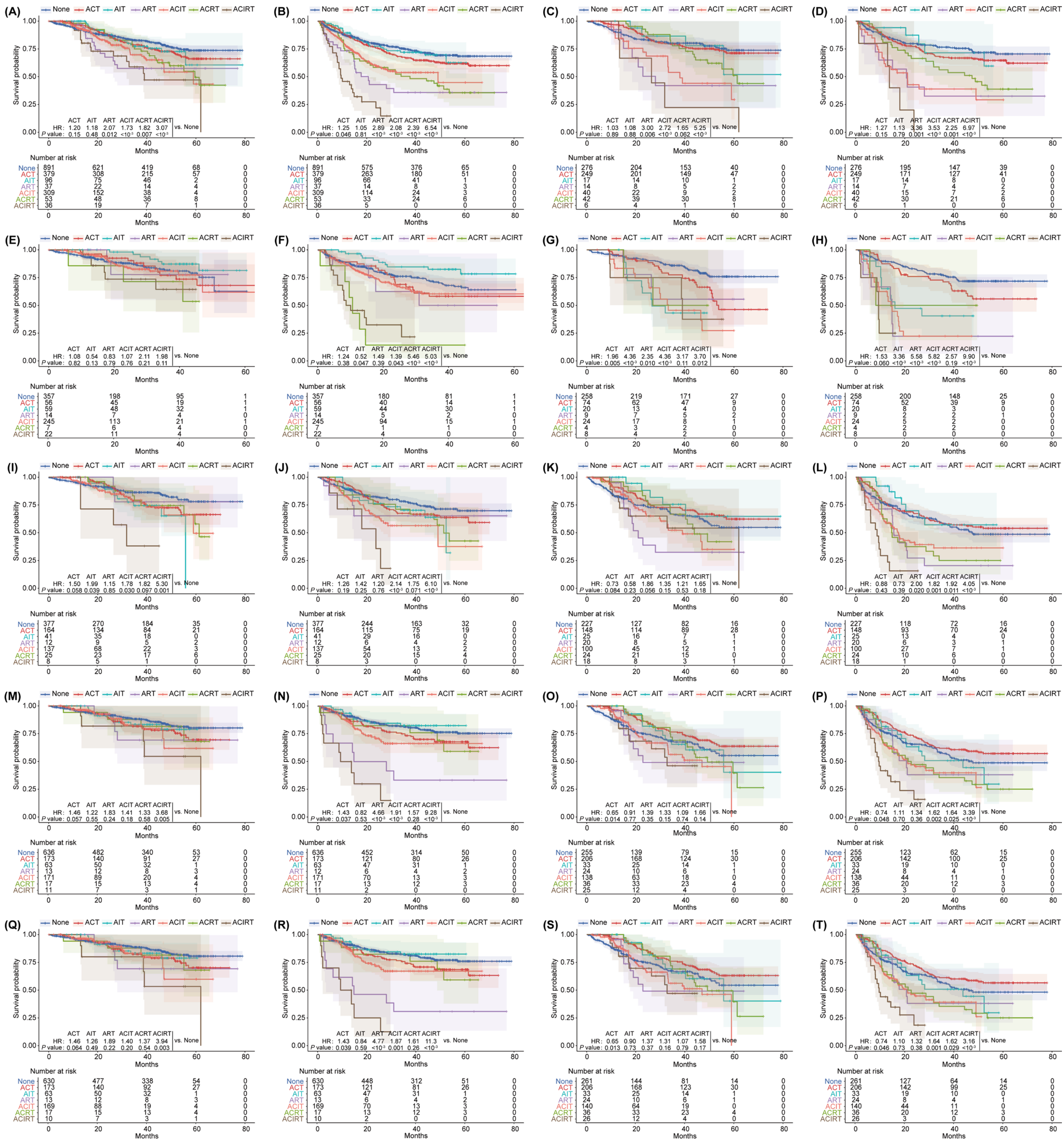

Figure S6. Overall survival (OS) and progression-free survival (PFS) by postoperative adjuvant therapy regimen.

A-B: OS (A) and PFS (B) for patients receiving different adjuvant therapies.

C-D: OS (C) and PFS (D) for patients who received NCT, by subsequent adjuvant therapies.

E-F: OS (E) and PFS (F) for patients who received NCIT, by subsequent adjuvant therapies.

G-H: OS (G) and PFS (H) for patients who received NCRT, by subsequent adjuvant therapies.

I-J: OS (I) and PFS (J) for patients with ypT 1-2 tumors, by subsequent adjuvant therapies.

K-L: OS (K) and PFS (L) for patients with ypT 3-4 tumors, by subsequent adjuvant therapies.

M-N: OS (M) and PFS (N) for patients with ypN 0 tumors, by subsequent adjuvant therapies.

O-P: OS (O) and PFS (P) for patients with ypN 1-3 tumors, by subsequent adjuvant therapies.

Q-R: OS (Q) and PFS (R) for patients with ypTNM I-II tumors, by subsequent adjuvant therapies.

S-T: OS (S) and PFS (T) for patients with ypTNM III-IV tumors, by subsequent adjuvant therapies.

Abbreviations: ACT, adjuvant chemotherapy; ACIRT, adjuvant chemo-immuno-radiotherapy; ACIT, adjuvant chemoimmunotherapy; ACRT, adjuvant chemoradiotherapy; AIT, adjuvant immunotherapy; ART, adjuvant radiotherapy; HR, hazard ratio.

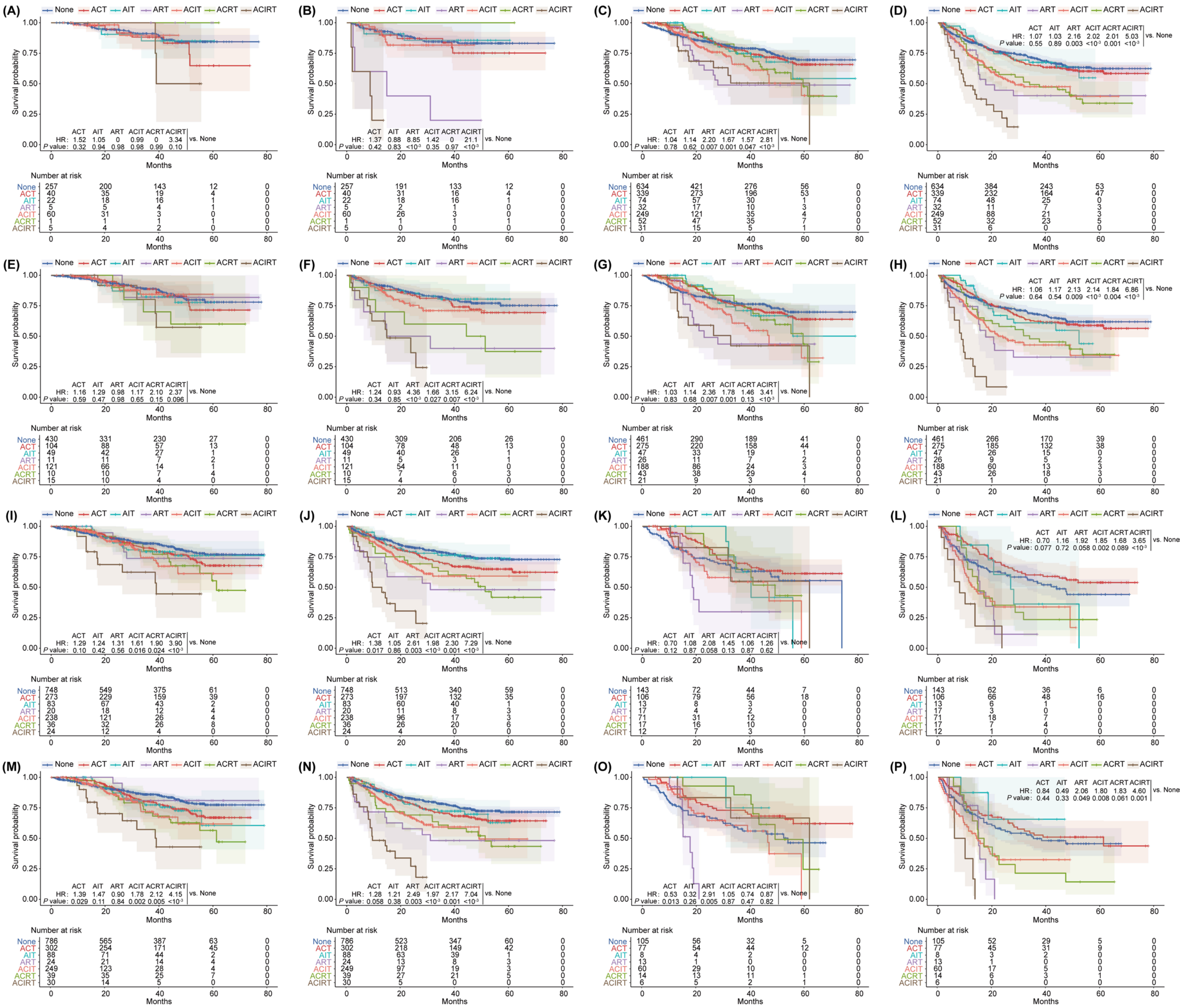

Figure S7. Overall survival (OS) and progression-free survival (PFS) by postoperative adjuvant therapy regimen.

A-B: OS (A) and PFS (B) for patients who achieved complete pathologic response (CPR), by subsequent adjuvant therapies.

C-D: OS (C) and PFS (D) for non-CPR patients, by subsequent adjuvant therapies.

E-F: OS (E) and PFS (F) for patients who achieved major pathological response (MPR), by subsequent adjuvant therapies.

G-H: OS (G) and PFS (H) for non-MPR patients, by subsequent adjuvant therapies.

I-J: OS (I) and PFS (J) for patients without lymphovascular invasion, by subsequent adjuvant therapies.

K-L: OS (K) and PFS (L) for patients with lymphovascular invasion, by subsequent adjuvant therapies.

M-N: OS (M) and PFS (N) for patients without perineural invasion, by subsequent adjuvant therapies.

O-P: OS (O) and PFS (P) for patients with perineural invasion, by subsequent adjuvant therapies.

Abbreviations: ACT, adjuvant chemotherapy; ACIRT, adjuvant chemo-immuno-radiotherapy; ACIT, adjuvant chemoimmunotherapy; ACRT, adjuvant chemoradiotherapy; AIT, adjuvant immunotherapy; ART, adjuvant radiotherapy; ADT, adjuvant therapy; CPR, Complete pathologic response; HR, hazard ratio; MPR, major pathological response.

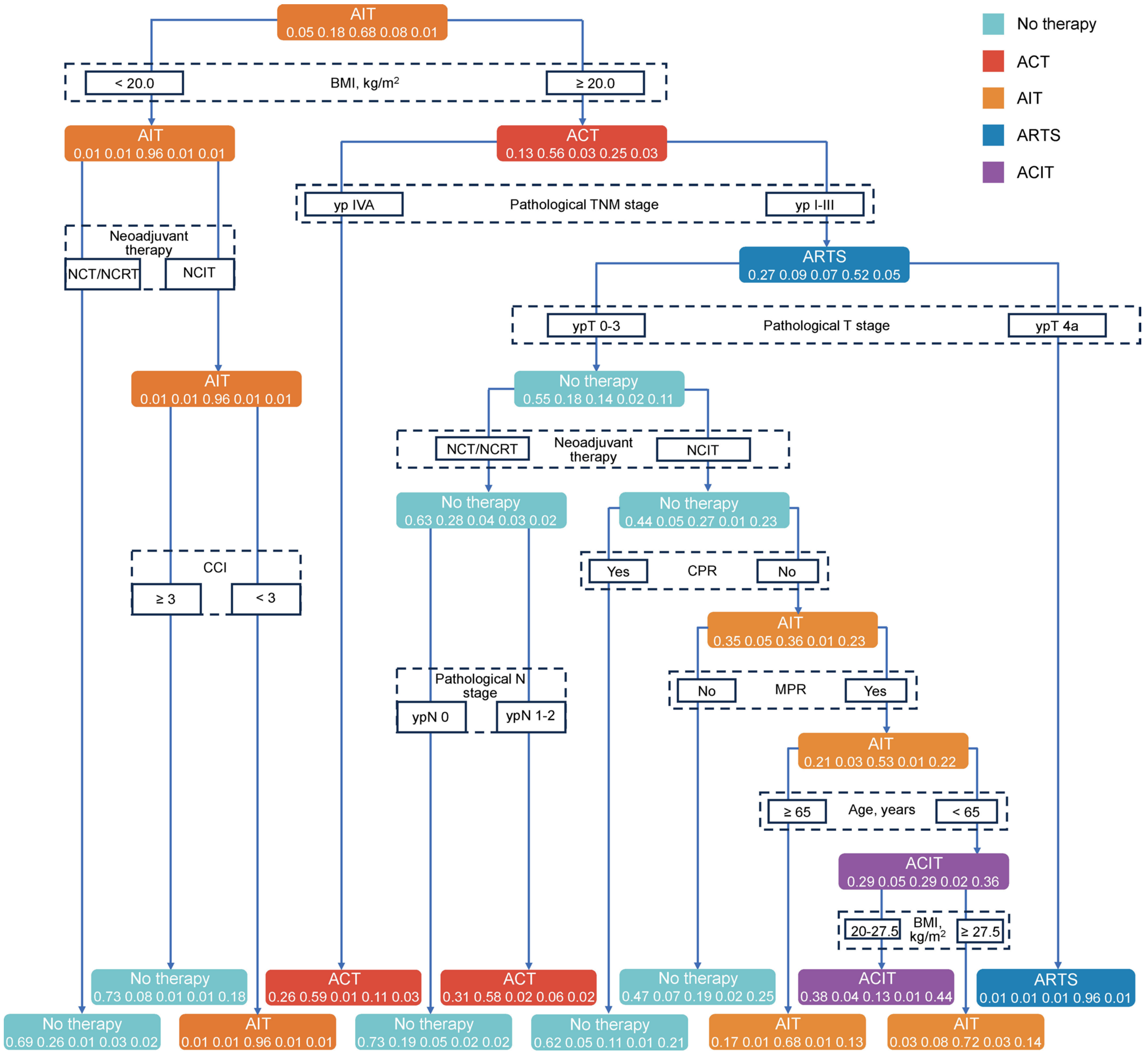

Figure S8. Optimal treatment rule (OTR) decision tree for selecting adjuvant therapies based on progression-free survival, using core subgroup variables as predictors. The five values in each box represent, from left to right, the probability of benefit from no adjuvant therapy, adjuvant chemotherapy (ACT), adjuvant immunotherapy (AIT), adjuvant radiotherapies (ARTS), and adjuvant chemoimmunotherapy (ACIT). ARTS comprises adjuvant radiotherapy alone, chemoradiotherapy, and chemo-immuno-radiotherapy.

Abbreviations: ACT, adjuvant chemotherapy; ACIT, adjuvant chemoimmunotherapy; AIT, adjuvant immunotherapy; ARTS, adjuvant radiotherapies; BMI, body mass index; CCI, Charlson comorbidity index; CPR, complete pathologic response; MPR, major pathological response; NCIT, neoadjuvant chemoimmunotherapy; NCRT, neoadjuvant chemoradiotherapy; NCT, neoadjuvant chemotherapy.
